# Supplementary material for: MPS1 promotes timely spindle bipolarization to prevent kinetochore-microtubule attachment errors in oocytes
Source: EMBO J. 2025 Jun 4;44(13):3794–823. doi: 10.1038/s44318-025-00461-w (PMC12214816; doi:10.1038/s44318-025-00461-w)
Supplement: Supplementary file 2 — Movie EV1 [file 44318_2025_461_MOESM2_ESM.zip › EMBOJ-2024-118908_MovieEV1.docx]

**Movie EV1: MPS1 inhibition delays spindle bipolarization and chromosome alignment** (related to Figures 1B and 4A).

Live imaging of oocytes expressing EGFP-MAP4 (spindle, green) and H2B-mCherry (chromosome, magenta), treated with proTAME. Time after NEBD (hours:minutes).
