# Supplementary material for: MPS1 promotes timely spindle bipolarization to prevent kinetochore-microtubule attachment errors in oocytes
Source: EMBO J. 2025 Jun 4;44(13):3794–823. doi: 10.1038/s44318-025-00461-w (PMC12214816; doi:10.1038/s44318-025-00461-w)
Supplement: Supplementary file 3 — Movie EV2 [file 44318_2025_461_MOESM3_ESM.zip › EMBOJ-2024-118908_MovieEV2.docx]

**Movie EV2: MPS1 inhibition impairs spindle bipolarization in NDC80-9D oocytes** (related to Figure 1D).

Live imaging of *Ndc80^f/f^ Zp3-Cre* oocytes expressing EGFP-MAP4 (spindle, green), H2B-mCherry (chromosome, magenta), and NDC80-WT/-9D, treated with reversine. Time after NEBD (hours:minutes).
