# Supplementary material for: MPS1 promotes timely spindle bipolarization to prevent kinetochore-microtubule attachment errors in oocytes
Source: EMBO J. 2025 Jun 4;44(13):3794–823. doi: 10.1038/s44318-025-00461-w (PMC12214816; doi:10.1038/s44318-025-00461-w)
Supplement: Supplementary file 4 — Movie EV3 [file 44318_2025_461_MOESM4_ESM.zip › EMBOJ-2024-118908_MovieEV3.docx]

**Movie EV3: PRC1 activity for spindle bipolarization depends on MPS1** (related to Figure 3C).

Live imaging of *Ndc80^f/f^ Zp3-Cre* oocytes expressing mNeonGreen-PRC1 (green) and H2B-mCherry (chromosome, magenta), treated with reversine. Time after NEBD (hours:minutes).
