# Supplementary figures and images for: MPS1 promotes timely spindle bipolarization to prevent kinetochore-microtubule attachment errors in oocytes
Source: EMBO J. 2025 Jun 4;44(13):3794–823. doi: 10.1038/s44318-025-00461-w (PMC12214816; doi:10.1038/s44318-025-00461-w)

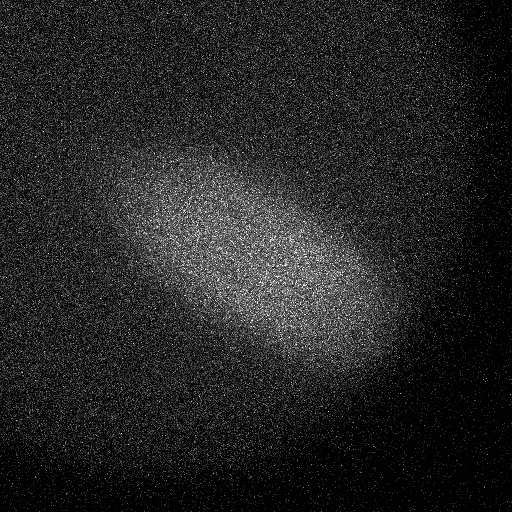

Supplement: Supplementary file 7 — Source data Fig. 1 [file 44318_2025_461_MOESM7_ESM.zip › Figure1/1A/Image_data/Left_bipolar.tif]

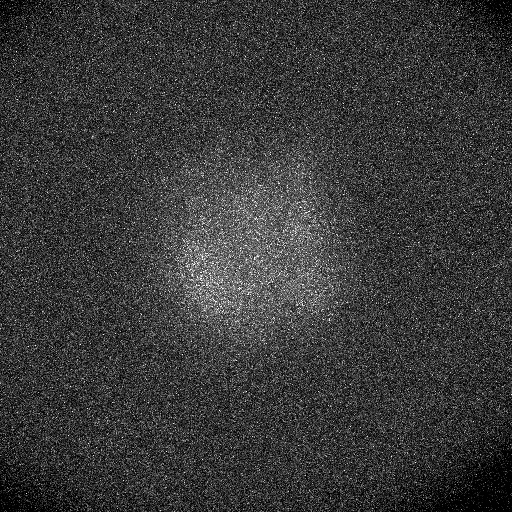

Supplement: Supplementary file 7 — Source data Fig. 1 [file 44318_2025_461_MOESM7_ESM.zip › Figure1/1B/Image_data/Control_0h.tif]

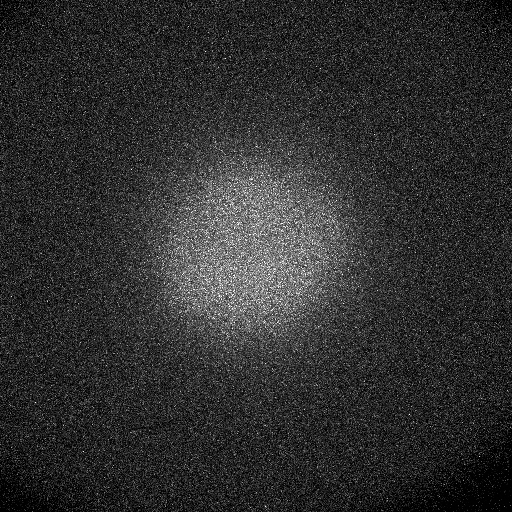

Supplement: Supplementary file 7 — Source data Fig. 1 [file 44318_2025_461_MOESM7_ESM.zip › Figure1/1B/Image_data/Control_2h.tif]

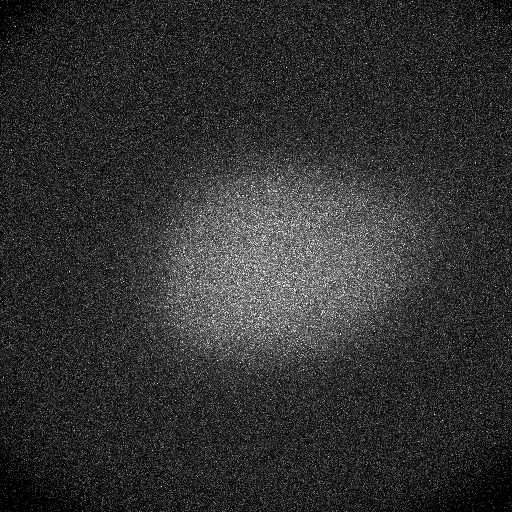

Supplement: Supplementary file 7 — Source data Fig. 1 [file 44318_2025_461_MOESM7_ESM.zip › Figure1/1B/Image_data/Control_4h.tif]

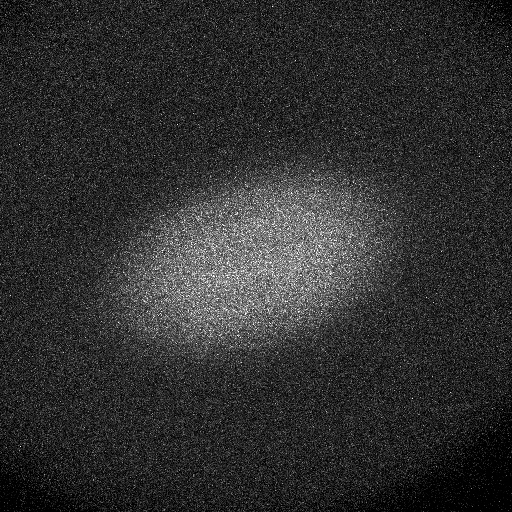

Supplement: Supplementary file 7 — Source data Fig. 1 [file 44318_2025_461_MOESM7_ESM.zip › Figure1/1B/Image_data/Control_6h.tif]

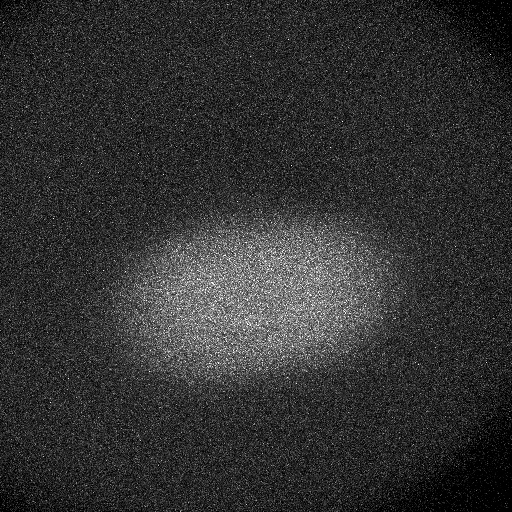

Supplement: Supplementary file 7 — Source data Fig. 1 [file 44318_2025_461_MOESM7_ESM.zip › Figure1/1B/Image_data/Control_8h.tif]

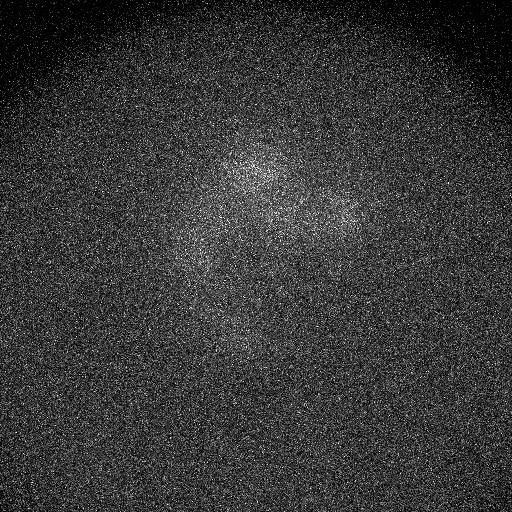

Supplement: Supplementary file 7 — Source data Fig. 1 [file 44318_2025_461_MOESM7_ESM.zip › Figure1/1B/Image_data/Reversine_0h.tif]

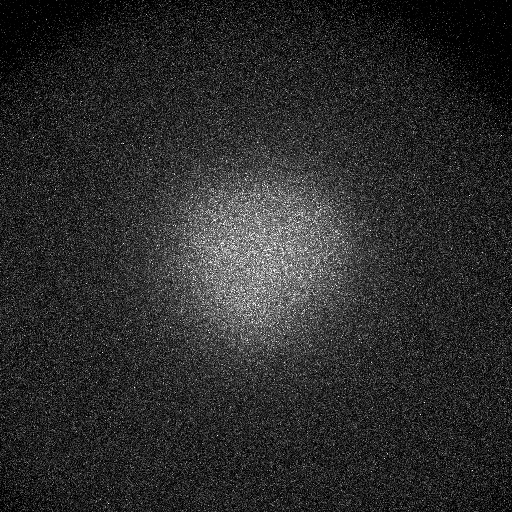

Supplement: Supplementary file 7 — Source data Fig. 1 [file 44318_2025_461_MOESM7_ESM.zip › Figure1/1B/Image_data/Reversine_2h.tif]

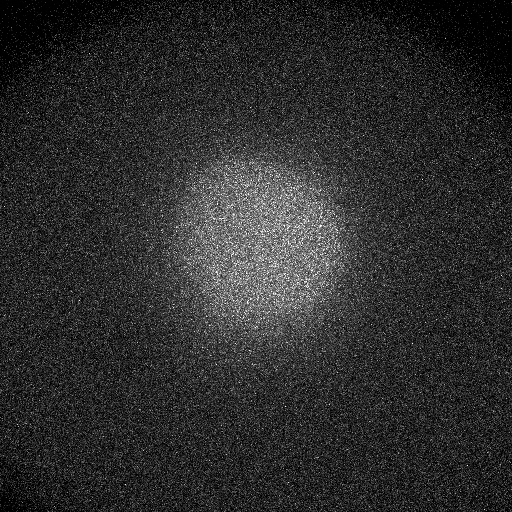

Supplement: Supplementary file 7 — Source data Fig. 1 [file 44318_2025_461_MOESM7_ESM.zip › Figure1/1B/Image_data/Reversine_4h.tif]

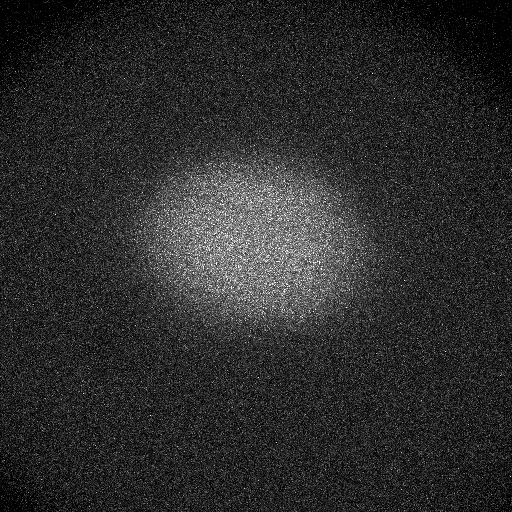

Supplement: Supplementary file 7 — Source data Fig. 1 [file 44318_2025_461_MOESM7_ESM.zip › Figure1/1B/Image_data/Reversine_6h.tif]

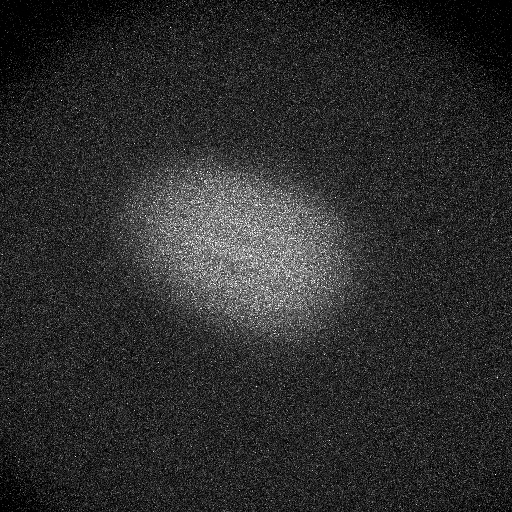

Supplement: Supplementary file 7 — Source data Fig. 1 [file 44318_2025_461_MOESM7_ESM.zip › Figure1/1B/Image_data/Reversine_8h.tif]

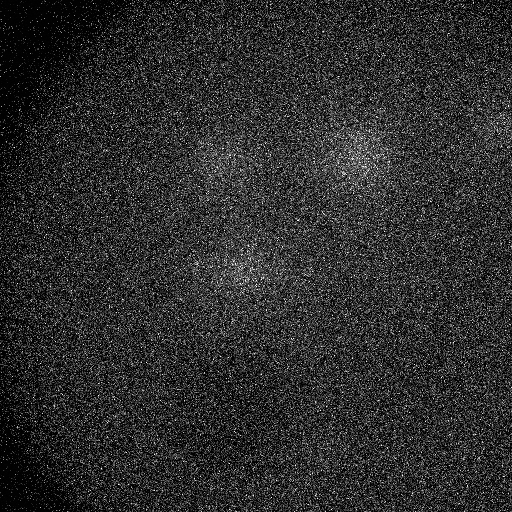

Supplement: Supplementary file 7 — Source data Fig. 1 [file 44318_2025_461_MOESM7_ESM.zip › Figure1/1C/Image_data/NDC80-9D_0h.tif]

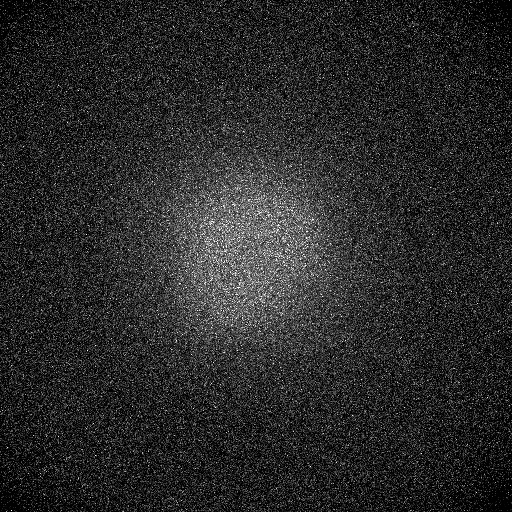

Supplement: Supplementary file 7 — Source data Fig. 1 [file 44318_2025_461_MOESM7_ESM.zip › Figure1/1C/Image_data/NDC80-9D_2h.tif]

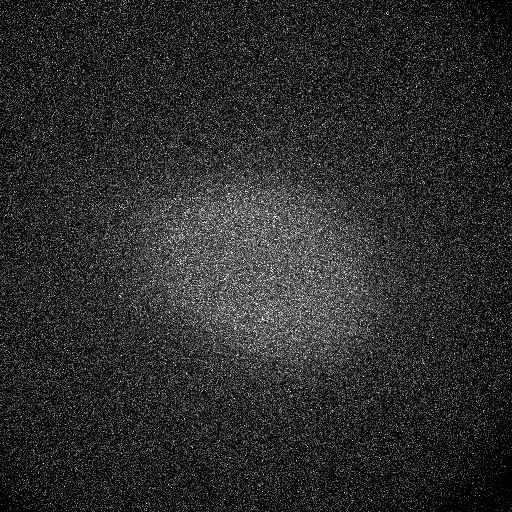

Supplement: Supplementary file 7 — Source data Fig. 1 [file 44318_2025_461_MOESM7_ESM.zip › Figure1/1C/Image_data/NDC80-9D_4h.tif]

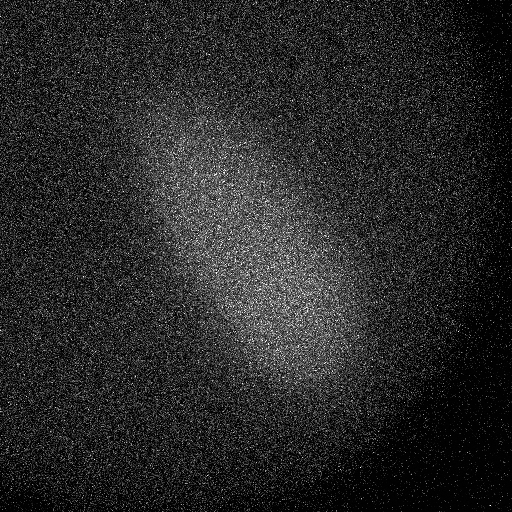

Supplement: Supplementary file 7 — Source data Fig. 1 [file 44318_2025_461_MOESM7_ESM.zip › Figure1/1C/Image_data/NDC80-9D_6h.tif]

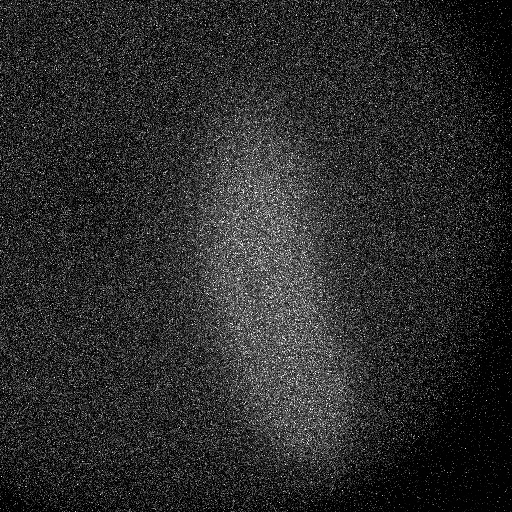

Supplement: Supplementary file 7 — Source data Fig. 1 [file 44318_2025_461_MOESM7_ESM.zip › Figure1/1C/Image_data/NDC80-9D_8h.tif]

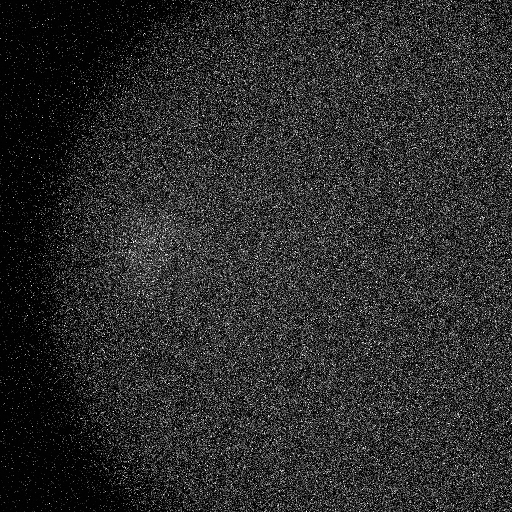

Supplement: Supplementary file 7 — Source data Fig. 1 [file 44318_2025_461_MOESM7_ESM.zip › Figure1/1C/Image_data/NDC80-WT_0h.tif]

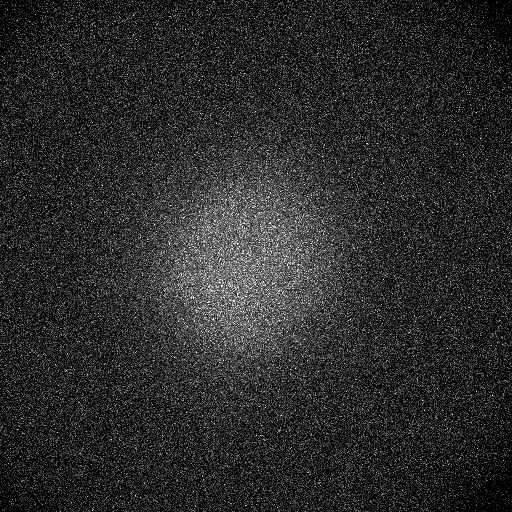

Supplement: Supplementary file 7 — Source data Fig. 1 [file 44318_2025_461_MOESM7_ESM.zip › Figure1/1C/Image_data/NDC80-WT_2h.tif]

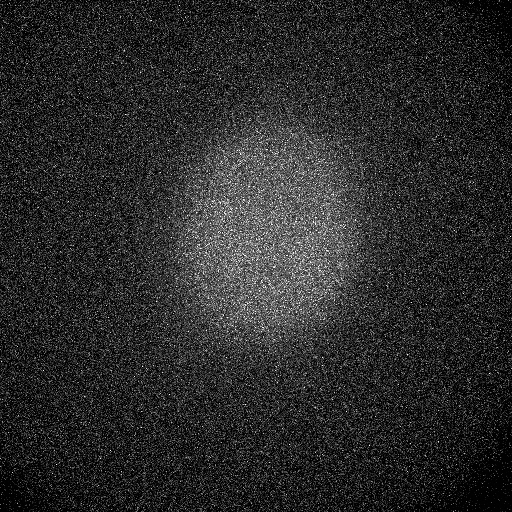

Supplement: Supplementary file 7 — Source data Fig. 1 [file 44318_2025_461_MOESM7_ESM.zip › Figure1/1C/Image_data/NDC80-WT_4h.tif]

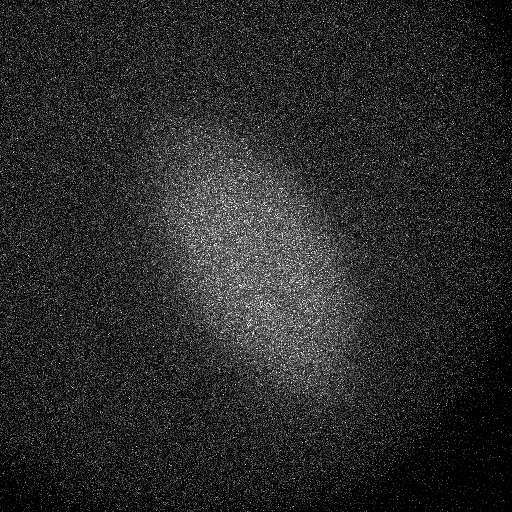

Supplement: Supplementary file 7 — Source data Fig. 1 [file 44318_2025_461_MOESM7_ESM.zip › Figure1/1C/Image_data/NDC80-WT_6h.tif]

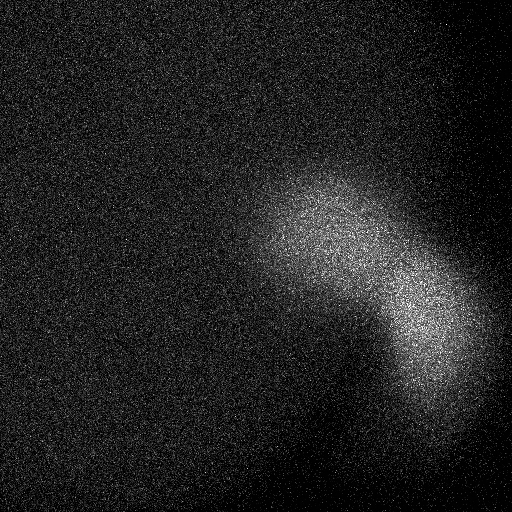

Supplement: Supplementary file 7 — Source data Fig. 1 [file 44318_2025_461_MOESM7_ESM.zip › Figure1/1C/Image_data/NDC80-WT_anaphase.tif]

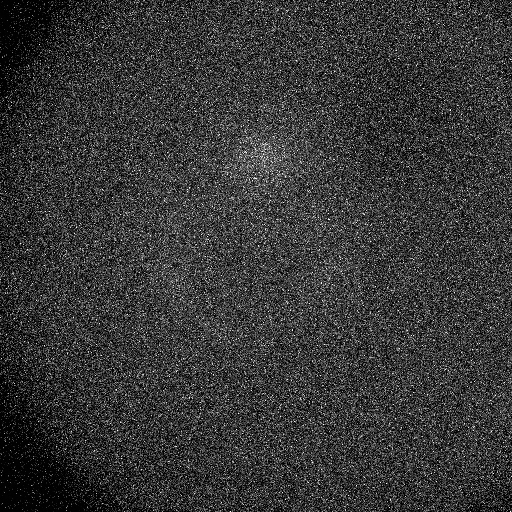

Supplement: Supplementary file 7 — Source data Fig. 1 [file 44318_2025_461_MOESM7_ESM.zip › Figure1/1D/Image_data/NDC80-9D_0h.tif]

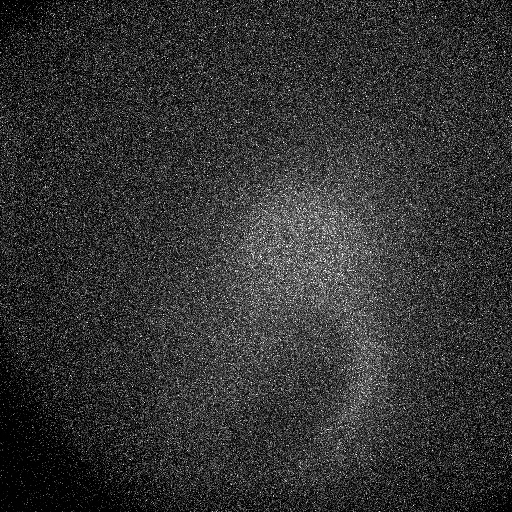

Supplement: Supplementary file 7 — Source data Fig. 1 [file 44318_2025_461_MOESM7_ESM.zip › Figure1/1D/Image_data/NDC80-9D_2h.tif]

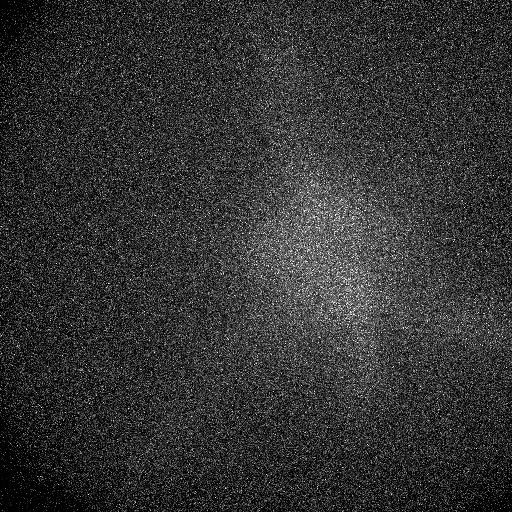

Supplement: Supplementary file 7 — Source data Fig. 1 [file 44318_2025_461_MOESM7_ESM.zip › Figure1/1D/Image_data/NDC80-9D_4h.tif]

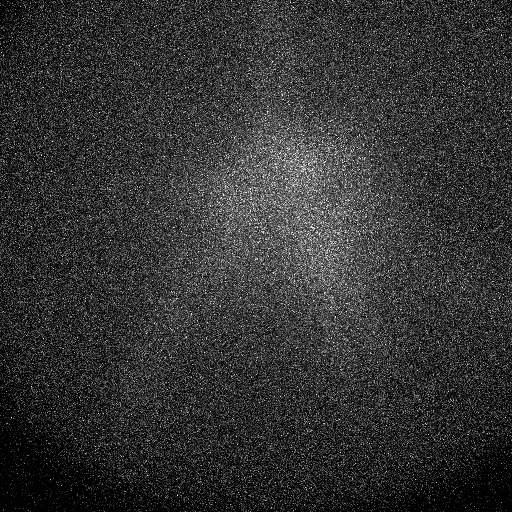

Supplement: Supplementary file 7 — Source data Fig. 1 [file 44318_2025_461_MOESM7_ESM.zip › Figure1/1D/Image_data/NDC80-9D_5h.tif]

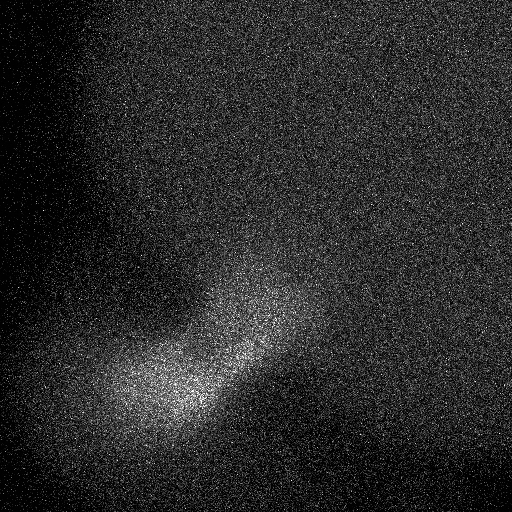

Supplement: Supplementary file 7 — Source data Fig. 1 [file 44318_2025_461_MOESM7_ESM.zip › Figure1/1D/Image_data/NDC80-9D_AnaI.tif]

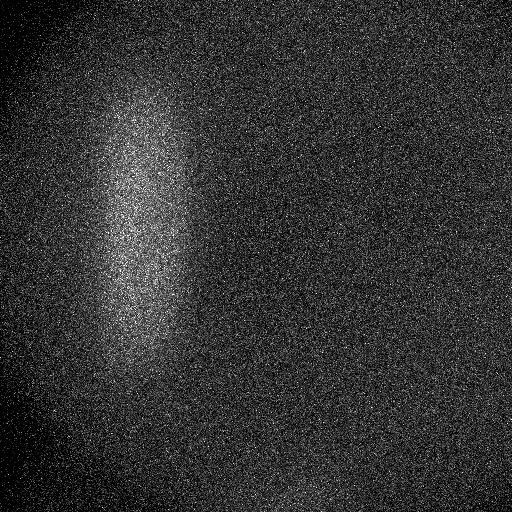

Supplement: Supplementary file 7 — Source data Fig. 1 [file 44318_2025_461_MOESM7_ESM.zip › Figure1/1D/Image_data/NDC80-9D_MetaII.tif]

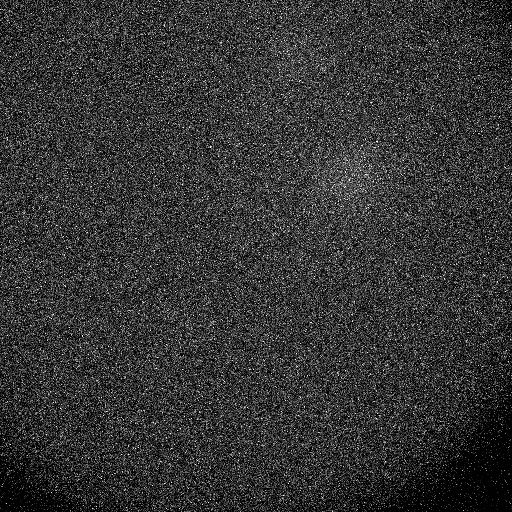

Supplement: Supplementary file 7 — Source data Fig. 1 [file 44318_2025_461_MOESM7_ESM.zip › Figure1/1D/Image_data/NDC80-WT_0h.tif]

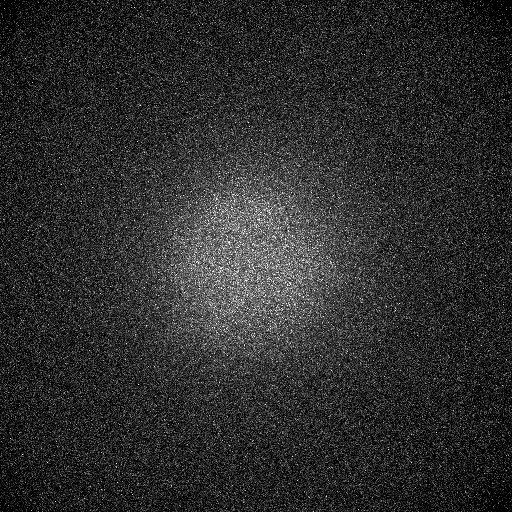

Supplement: Supplementary file 7 — Source data Fig. 1 [file 44318_2025_461_MOESM7_ESM.zip › Figure1/1D/Image_data/NDC80-WT_2h.tif]

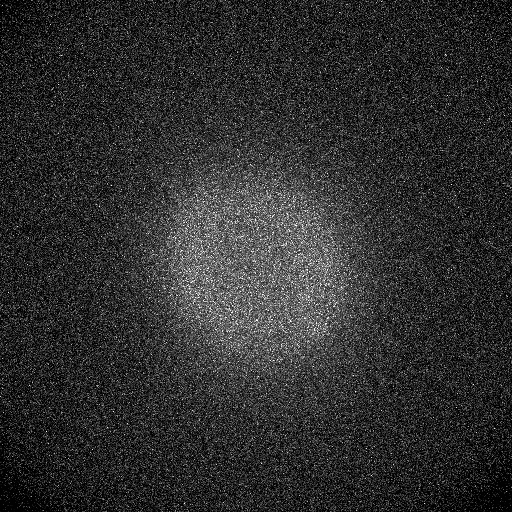

Supplement: Supplementary file 7 — Source data Fig. 1 [file 44318_2025_461_MOESM7_ESM.zip › Figure1/1D/Image_data/NDC80-WT_4h.tif]

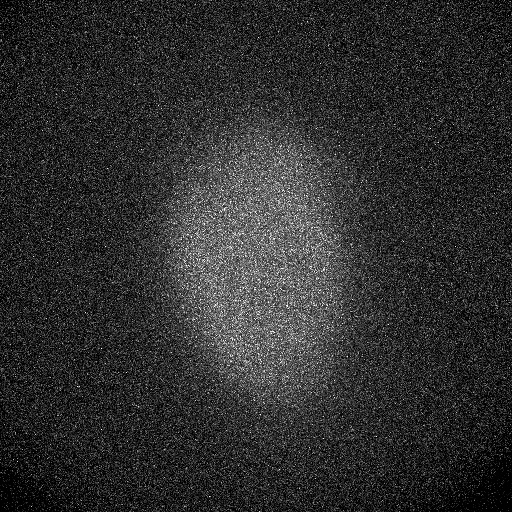

Supplement: Supplementary file 7 — Source data Fig. 1 [file 44318_2025_461_MOESM7_ESM.zip › Figure1/1D/Image_data/NDC80-WT_5h.tif]

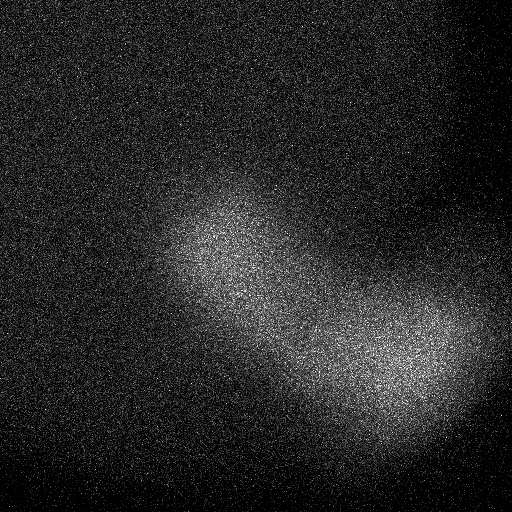

Supplement: Supplementary file 7 — Source data Fig. 1 [file 44318_2025_461_MOESM7_ESM.zip › Figure1/1D/Image_data/NDC80-WT_AnaI.tif]

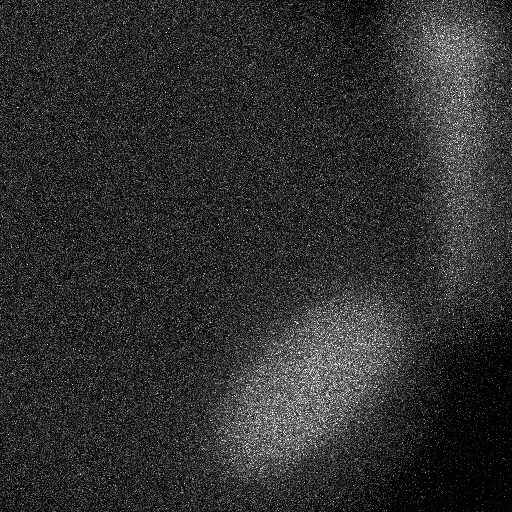

Supplement: Supplementary file 7 — Source data Fig. 1 [file 44318_2025_461_MOESM7_ESM.zip › Figure1/1D/Image_data/NDC80-WT_MetaII.tif]

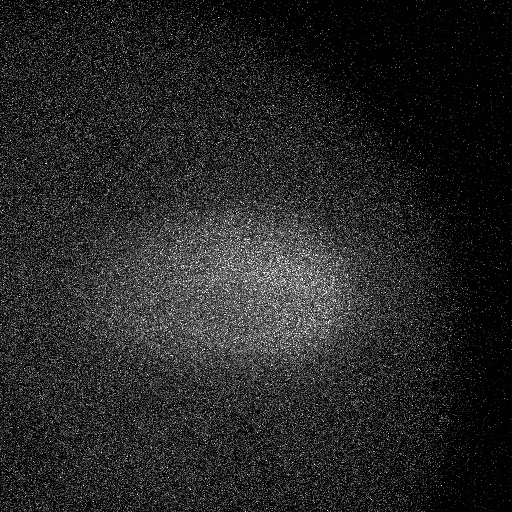

Supplement: Supplementary file 8 — Source data Fig. 2 [file 44318_2025_461_MOESM8_ESM.zip › Figure2/Fig2B/Image_data/Control.tif]

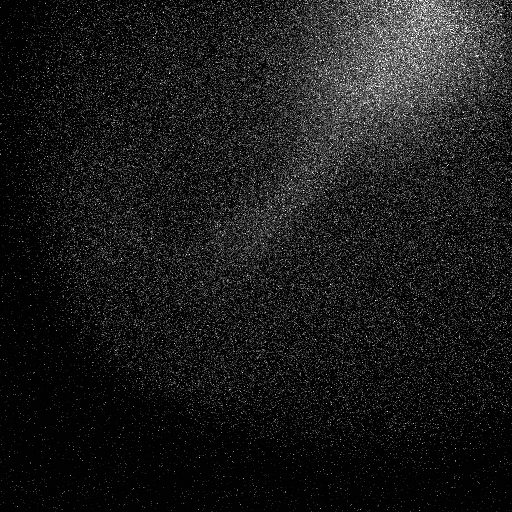

Supplement: Supplementary file 8 — Source data Fig. 2 [file 44318_2025_461_MOESM8_ESM.zip › Figure2/Fig2B/Image_data/Reversine.tif]

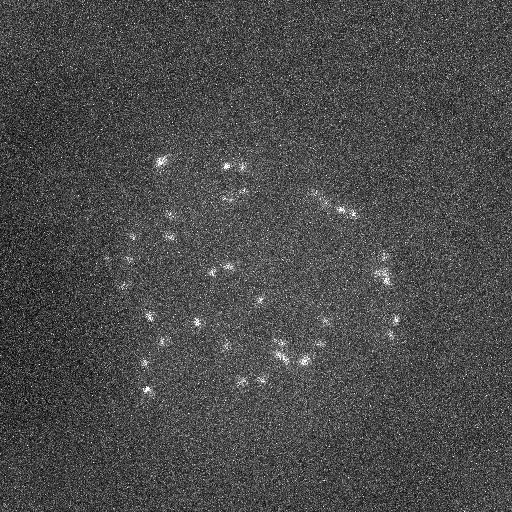

Supplement: Supplementary file 8 — Source data Fig. 2 [file 44318_2025_461_MOESM8_ESM.zip › Figure2/Fig2C/Image_data/Control.tif]

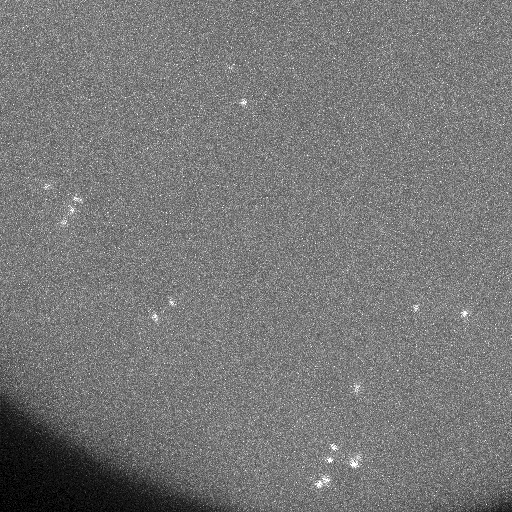

Supplement: Supplementary file 8 — Source data Fig. 2 [file 44318_2025_461_MOESM8_ESM.zip › Figure2/Fig2C/Image_data/Reversine.tif]

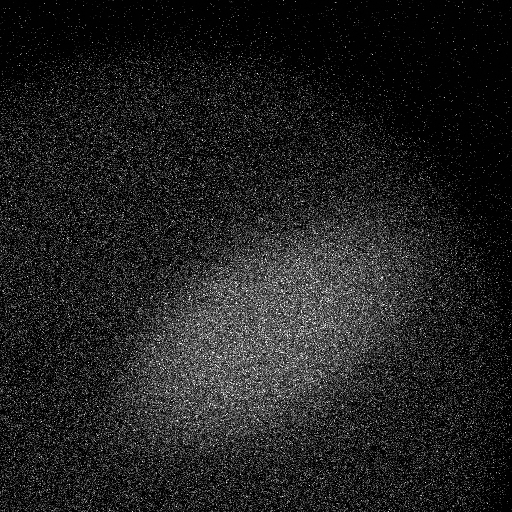

Supplement: Supplementary file 8 — Source data Fig. 2 [file 44318_2025_461_MOESM8_ESM.zip › Figure2/Fig2D/Image_data/Control.tif]

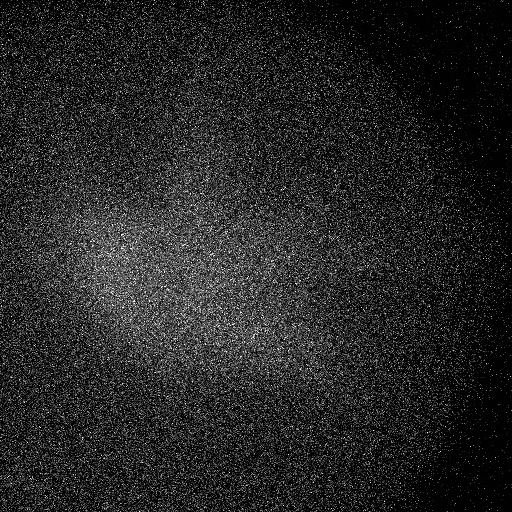

Supplement: Supplementary file 8 — Source data Fig. 2 [file 44318_2025_461_MOESM8_ESM.zip › Figure2/Fig2D/Image_data/Reversine.tif]

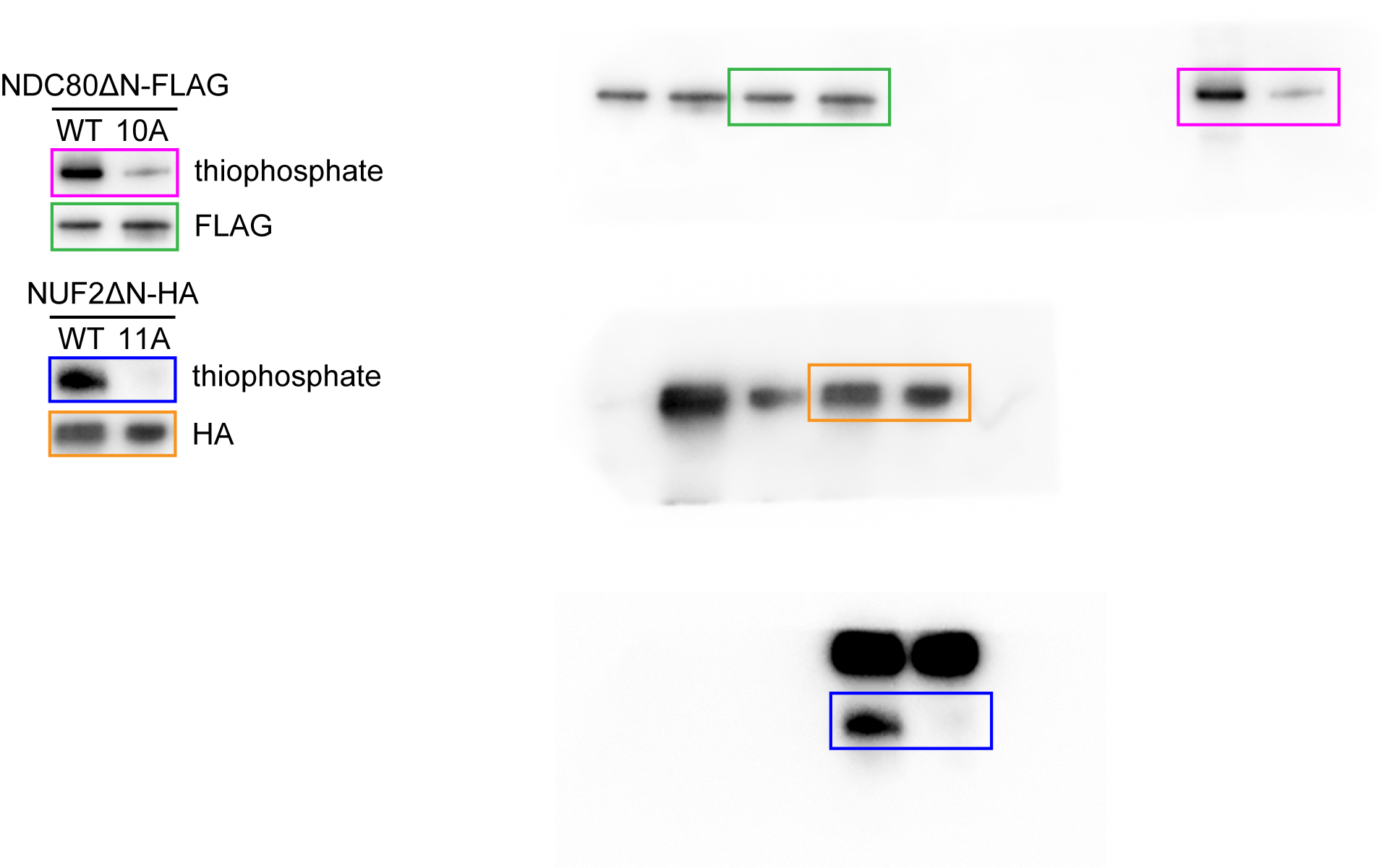

Supplement: Supplementary file 8 — Source data Fig. 2 [file 44318_2025_461_MOESM8_ESM.zip › Figure2/Fig2E/Image_data/2E.tif]

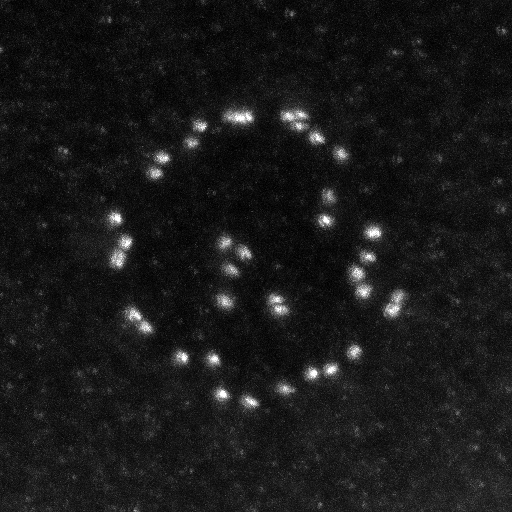

Supplement: Supplementary file 8 — Source data Fig. 2 [file 44318_2025_461_MOESM8_ESM.zip › Figure2/Fig2F/Image_data/Control.tif]

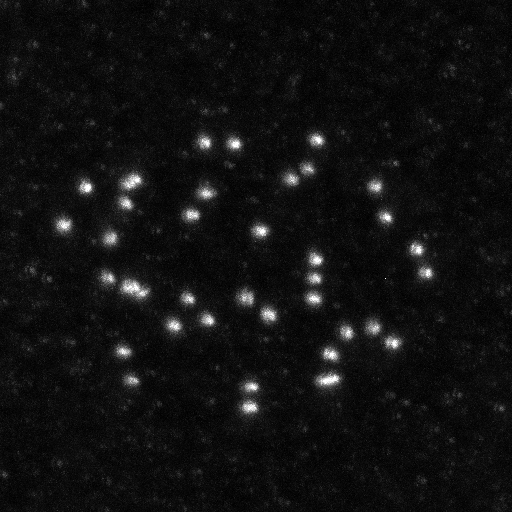

Supplement: Supplementary file 8 — Source data Fig. 2 [file 44318_2025_461_MOESM8_ESM.zip › Figure2/Fig2F/Image_data/Reversine.tif]

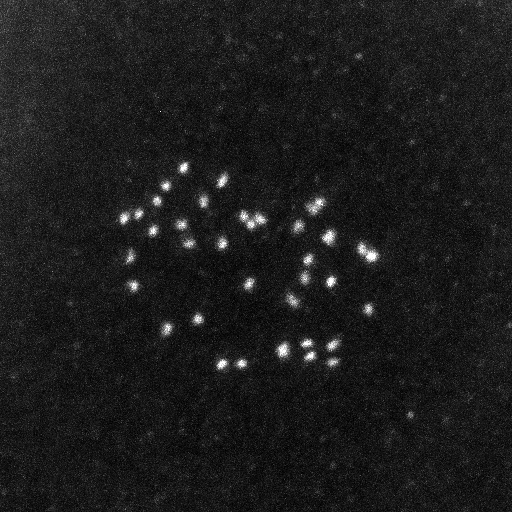

Supplement: Supplementary file 8 — Source data Fig. 2 [file 44318_2025_461_MOESM8_ESM.zip › Figure2/Fig2G/Image_data/2h.tif]

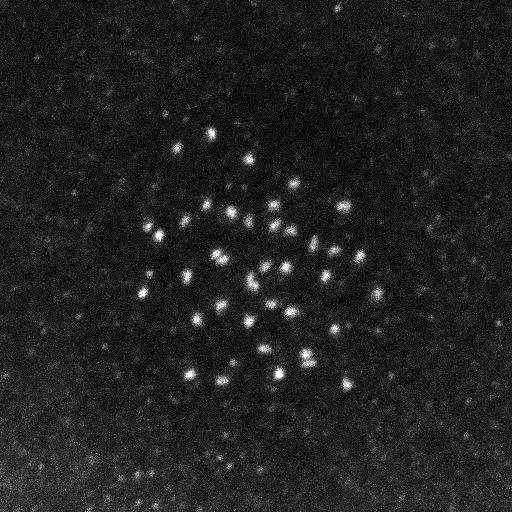

Supplement: Supplementary file 8 — Source data Fig. 2 [file 44318_2025_461_MOESM8_ESM.zip › Figure2/Fig2G/Image_data/4h.tif]

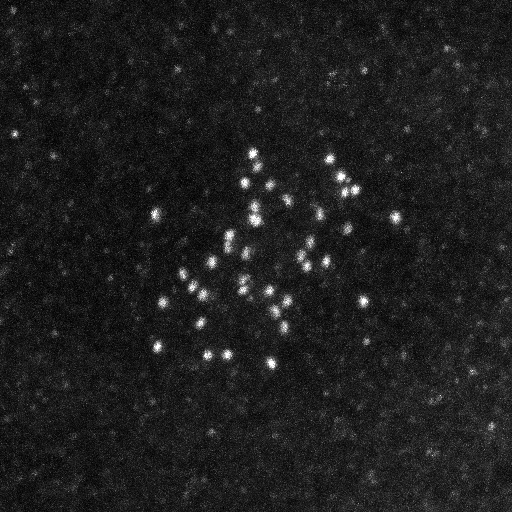

Supplement: Supplementary file 8 — Source data Fig. 2 [file 44318_2025_461_MOESM8_ESM.zip › Figure2/Fig2G/Image_data/6h.tif]

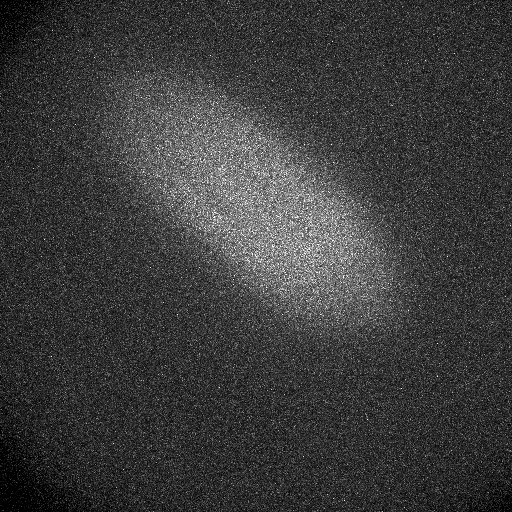

Supplement: Supplementary file 8 — Source data Fig. 2 [file 44318_2025_461_MOESM8_ESM.zip › Figure2/Fig2H/Image_data/10A11A.tif]

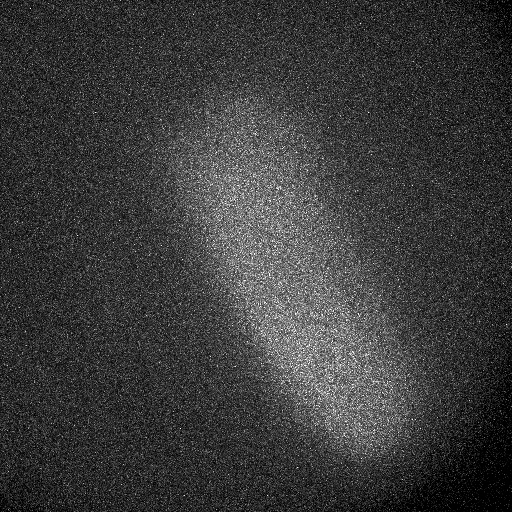

Supplement: Supplementary file 8 — Source data Fig. 2 [file 44318_2025_461_MOESM8_ESM.zip › Figure2/Fig2H/Image_data/WTWT.tif]

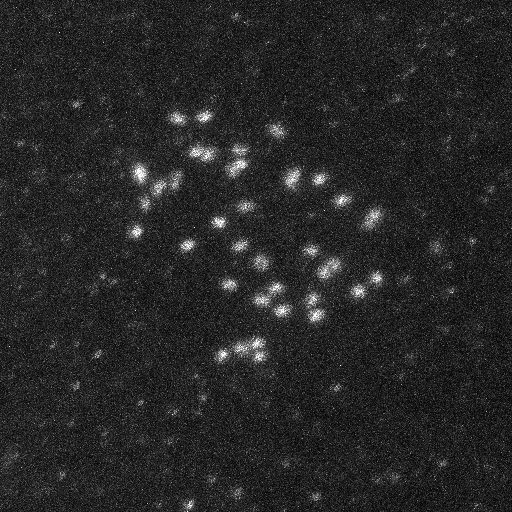

Supplement: Supplementary file 9 — Source data Fig. 3 [file 44318_2025_461_MOESM9_ESM.zip › Figure3/Fig3A/Image_data/Control.tif]

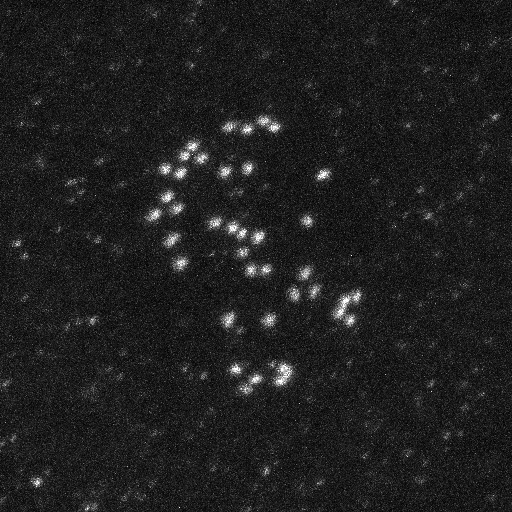

Supplement: Supplementary file 9 — Source data Fig. 3 [file 44318_2025_461_MOESM9_ESM.zip › Figure3/Fig3A/Image_data/Reversine.tif]

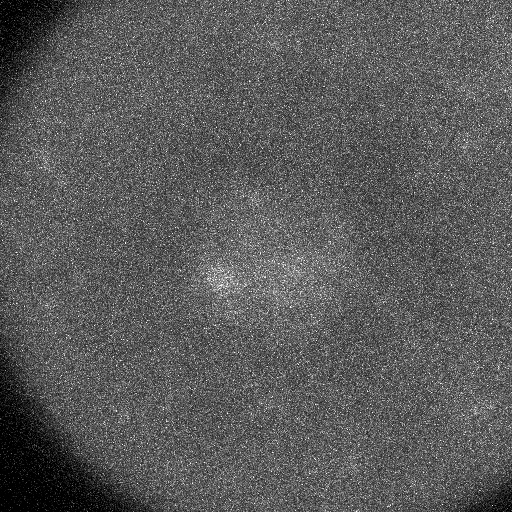

Supplement: Supplementary file 9 — Source data Fig. 3 [file 44318_2025_461_MOESM9_ESM.zip › Figure3/Fig3C/Image_data/Control_0h.tif]

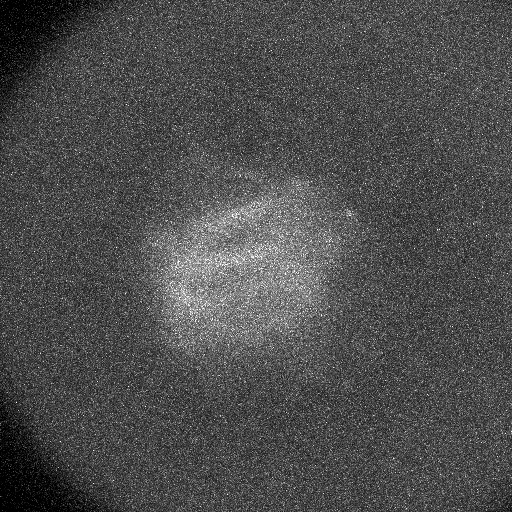

Supplement: Supplementary file 9 — Source data Fig. 3 [file 44318_2025_461_MOESM9_ESM.zip › Figure3/Fig3C/Image_data/Control_2h.tif]

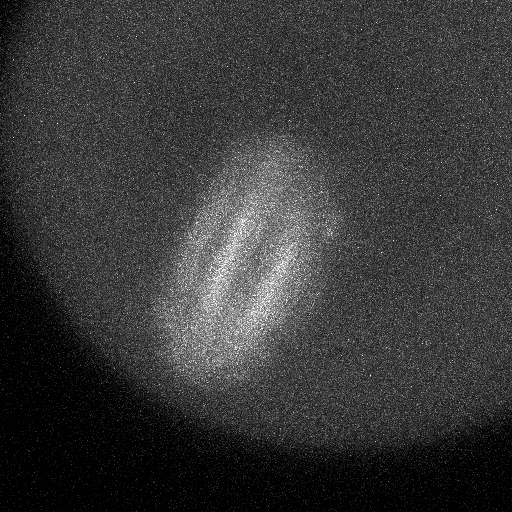

Supplement: Supplementary file 9 — Source data Fig. 3 [file 44318_2025_461_MOESM9_ESM.zip › Figure3/Fig3C/Image_data/Control_4h.tif]

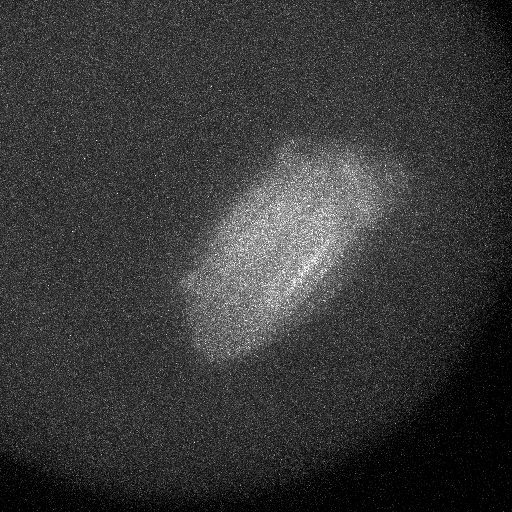

Supplement: Supplementary file 9 — Source data Fig. 3 [file 44318_2025_461_MOESM9_ESM.zip › Figure3/Fig3C/Image_data/Control_5h.tif]

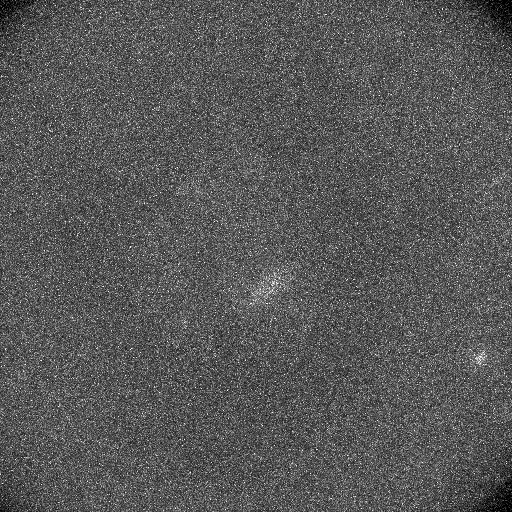

Supplement: Supplementary file 9 — Source data Fig. 3 [file 44318_2025_461_MOESM9_ESM.zip › Figure3/Fig3C/Image_data/Reversine_0h.tif]

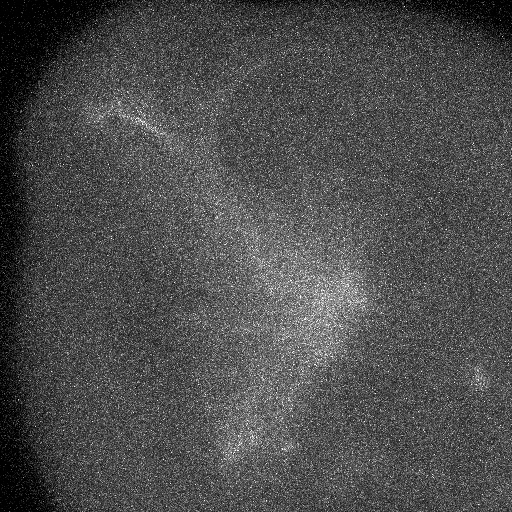

Supplement: Supplementary file 9 — Source data Fig. 3 [file 44318_2025_461_MOESM9_ESM.zip › Figure3/Fig3C/Image_data/Reversine_2h.tif]

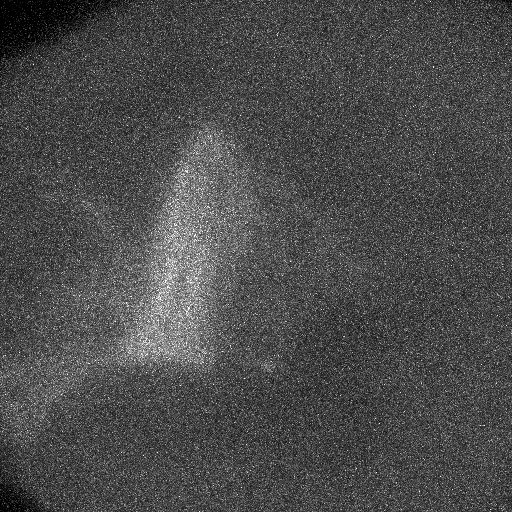

Supplement: Supplementary file 9 — Source data Fig. 3 [file 44318_2025_461_MOESM9_ESM.zip › Figure3/Fig3C/Image_data/Reversine_4h.tif]

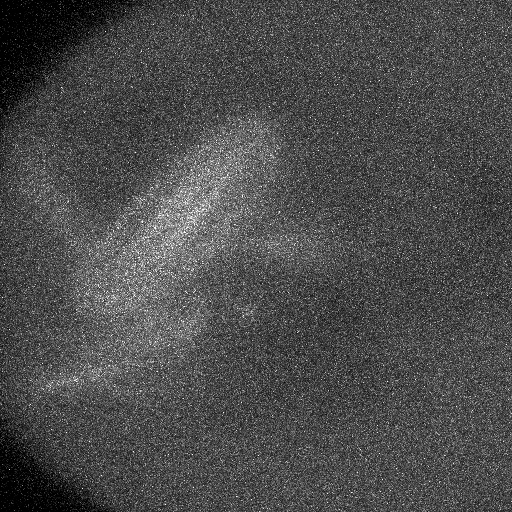

Supplement: Supplementary file 9 — Source data Fig. 3 [file 44318_2025_461_MOESM9_ESM.zip › Figure3/Fig3C/Image_data/Reversine_5h.tif]

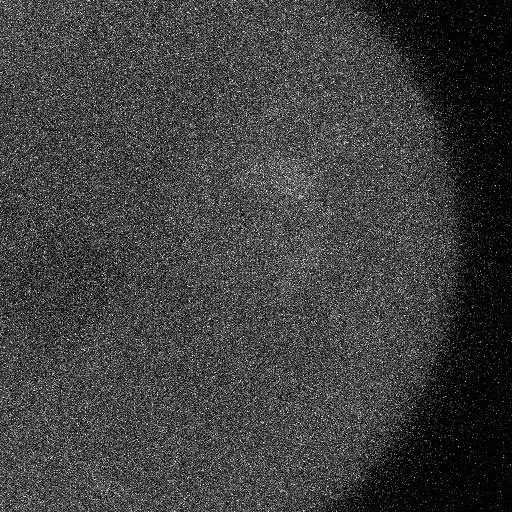

Supplement: Supplementary file 9 — Source data Fig. 3 [file 44318_2025_461_MOESM9_ESM.zip › Figure3/Fig3D/Image_data/PRC1-2A_0h.tif]

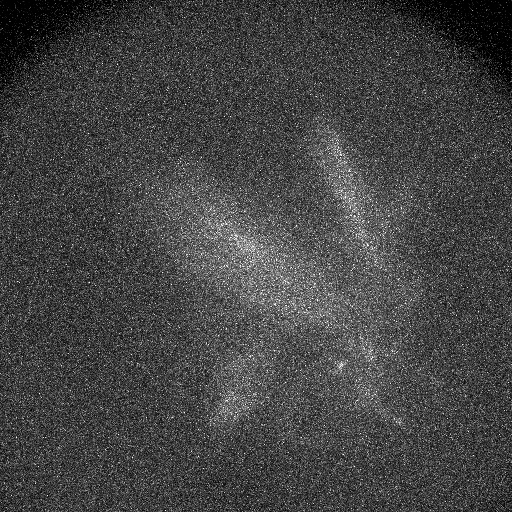

Supplement: Supplementary file 9 — Source data Fig. 3 [file 44318_2025_461_MOESM9_ESM.zip › Figure3/Fig3D/Image_data/PRC1-2A_2h.tif]

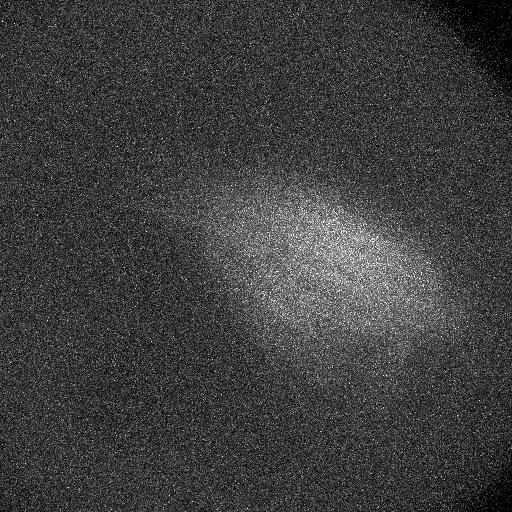

Supplement: Supplementary file 9 — Source data Fig. 3 [file 44318_2025_461_MOESM9_ESM.zip › Figure3/Fig3D/Image_data/PRC1-2A_4h.tif]

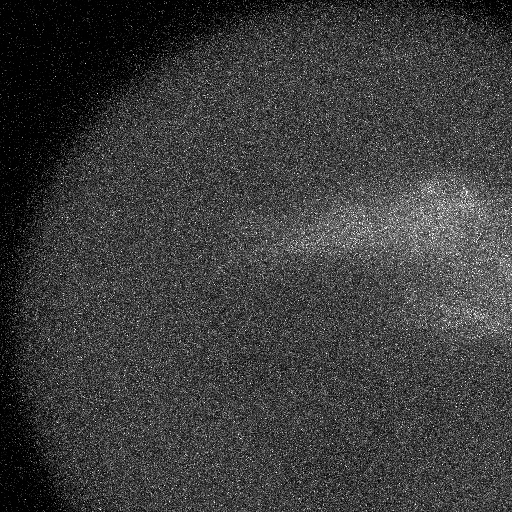

Supplement: Supplementary file 9 — Source data Fig. 3 [file 44318_2025_461_MOESM9_ESM.zip › Figure3/Fig3D/Image_data/PRC1-2A_5h.tif]

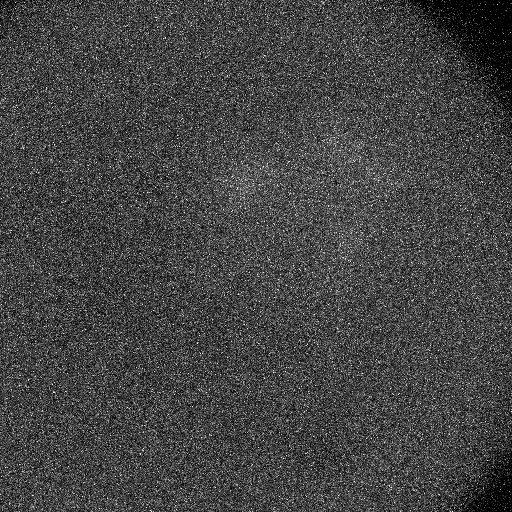

Supplement: Supplementary file 9 — Source data Fig. 3 [file 44318_2025_461_MOESM9_ESM.zip › Figure3/Fig3D/Image_data/WT_0h.tif]

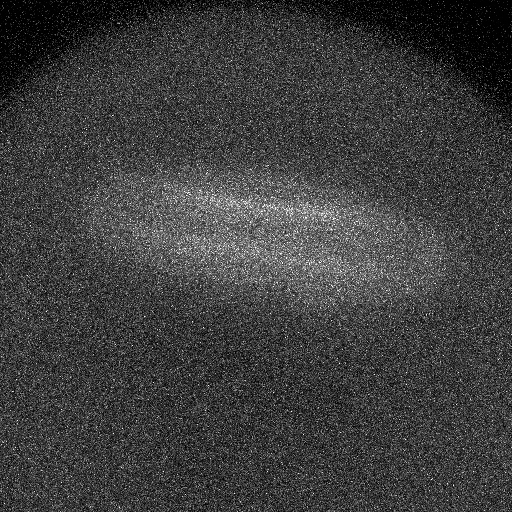

Supplement: Supplementary file 9 — Source data Fig. 3 [file 44318_2025_461_MOESM9_ESM.zip › Figure3/Fig3D/Image_data/WT_2h.tif]

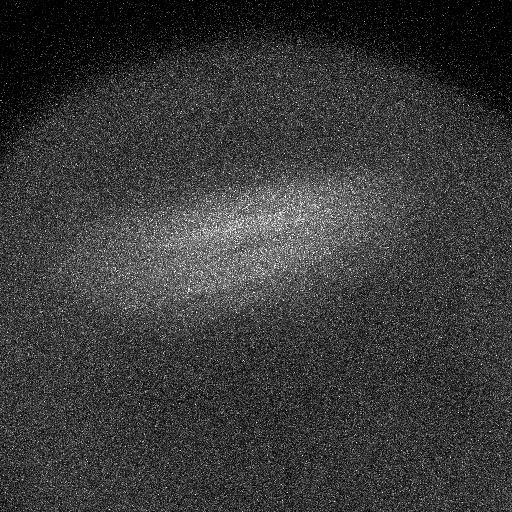

Supplement: Supplementary file 9 — Source data Fig. 3 [file 44318_2025_461_MOESM9_ESM.zip › Figure3/Fig3D/Image_data/WT_4h.tif]

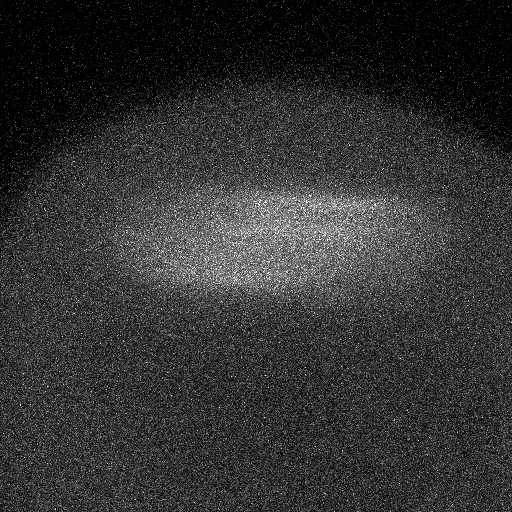

Supplement: Supplementary file 9 — Source data Fig. 3 [file 44318_2025_461_MOESM9_ESM.zip › Figure3/Fig3D/Image_data/WT_5h.tif]

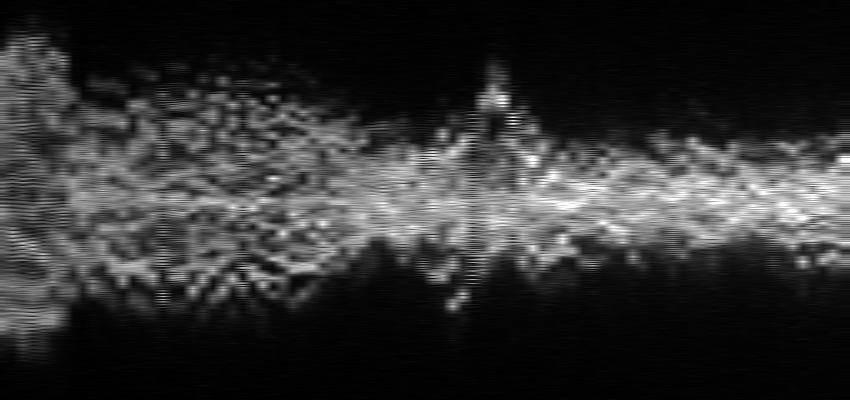

Supplement: Supplementary file 10 — Source data Fig. 4 [file 44318_2025_461_MOESM10_ESM.zip › Figure4/Fig4A/Image_data/Control.tif]

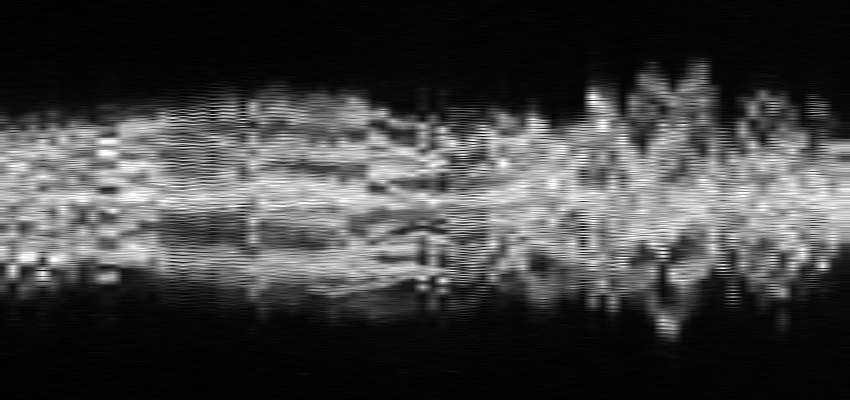

Supplement: Supplementary file 10 — Source data Fig. 4 [file 44318_2025_461_MOESM10_ESM.zip › Figure4/Fig4A/Image_data/Reversine.tif]

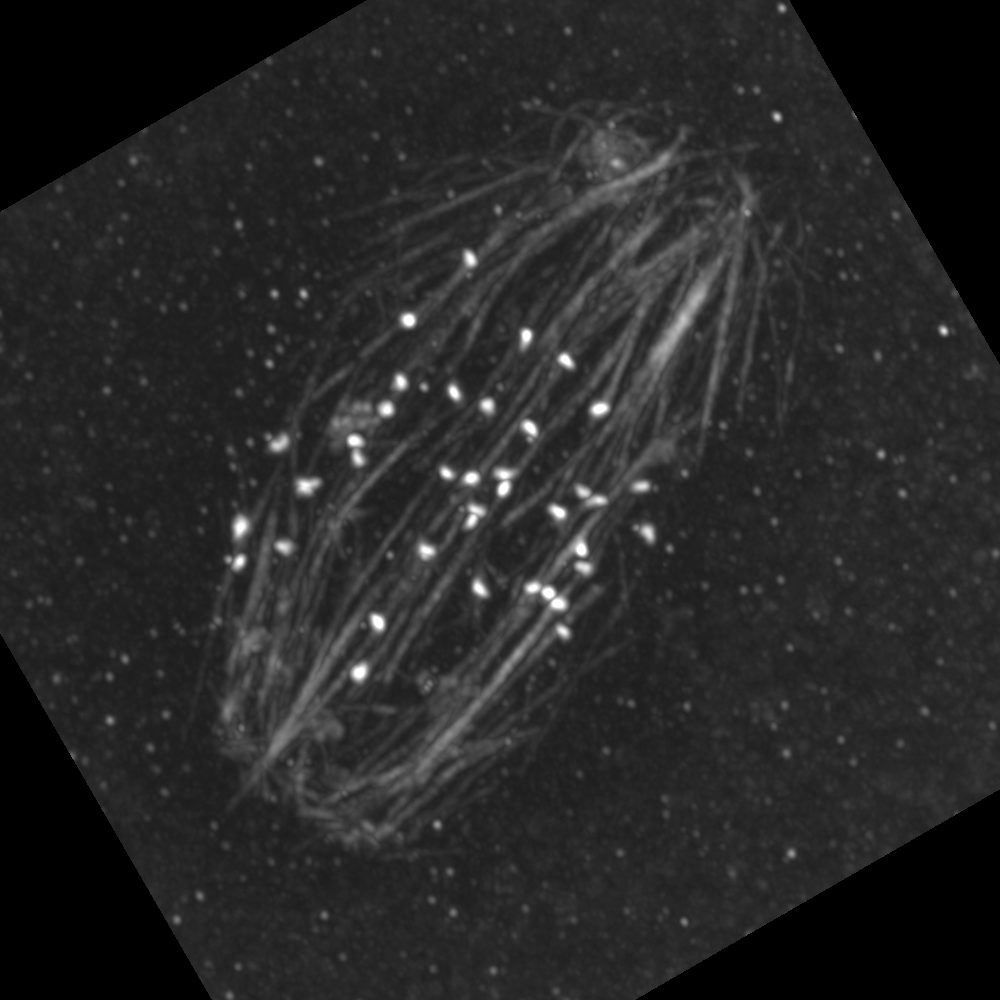

Supplement: Supplementary file 10 — Source data Fig. 4 [file 44318_2025_461_MOESM10_ESM.zip › Figure4/Fig4B/Image_data/Control.tif]

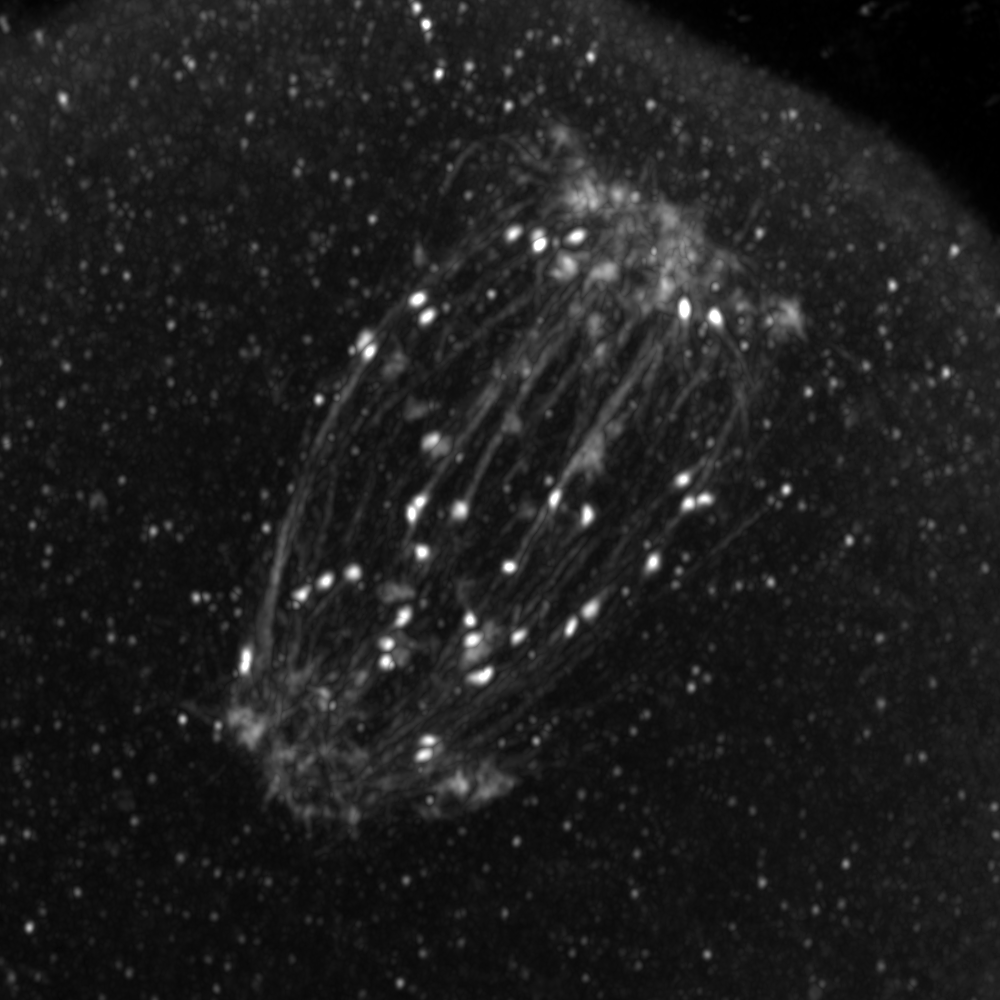

Supplement: Supplementary file 10 — Source data Fig. 4 [file 44318_2025_461_MOESM10_ESM.zip › Figure4/Fig4B/Image_data/Reversine.tif]

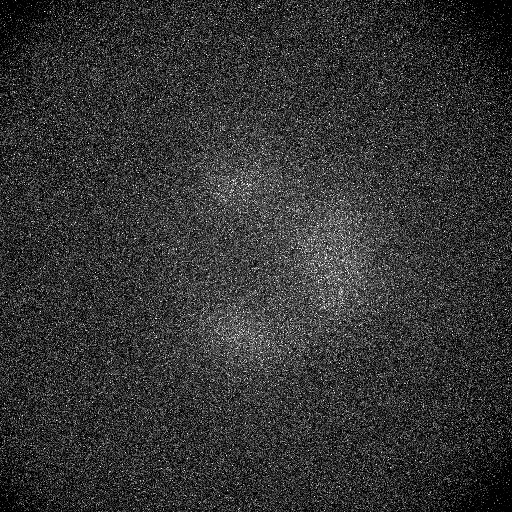

Supplement: Supplementary file 10 — Source data Fig. 4 [file 44318_2025_461_MOESM10_ESM.zip › Figure4/Fig4C/Image_data/+KifC1_0h.tif]

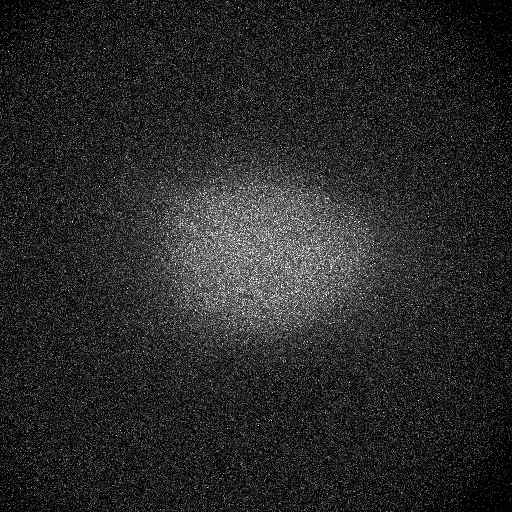

Supplement: Supplementary file 10 — Source data Fig. 4 [file 44318_2025_461_MOESM10_ESM.zip › Figure4/Fig4C/Image_data/+KifC1_2h.tif]

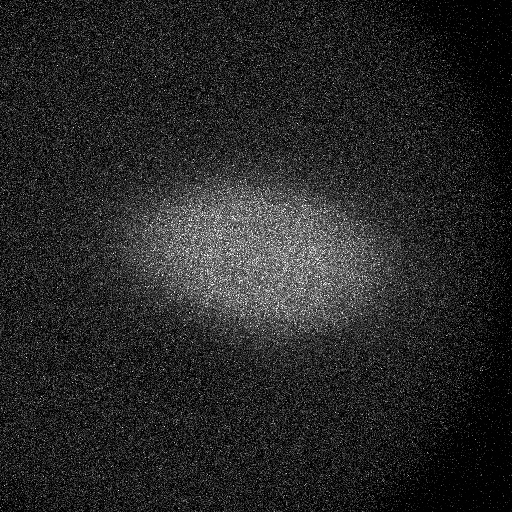

Supplement: Supplementary file 10 — Source data Fig. 4 [file 44318_2025_461_MOESM10_ESM.zip › Figure4/Fig4C/Image_data/+KifC1_4h.tif]

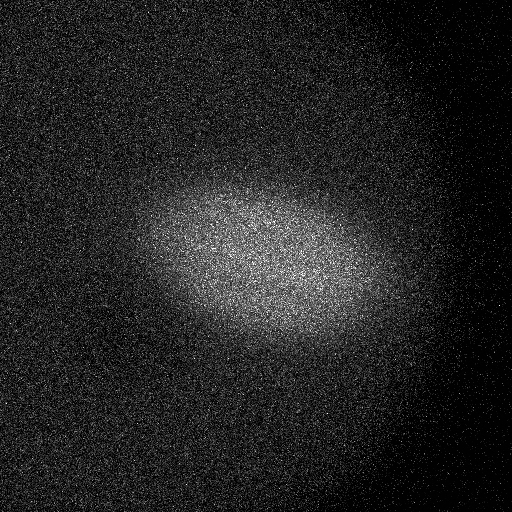

Supplement: Supplementary file 10 — Source data Fig. 4 [file 44318_2025_461_MOESM10_ESM.zip › Figure4/Fig4C/Image_data/+KifC1_6h.tif]

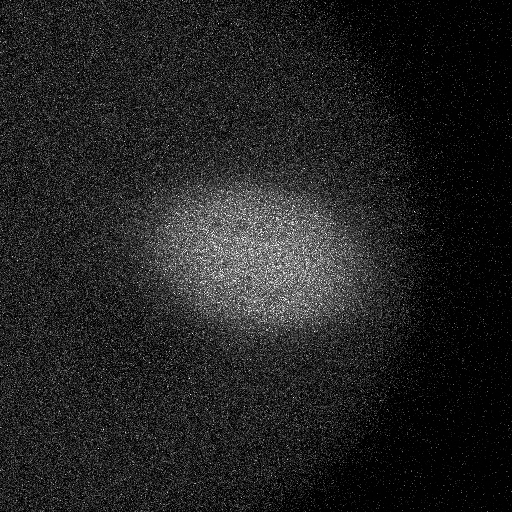

Supplement: Supplementary file 10 — Source data Fig. 4 [file 44318_2025_461_MOESM10_ESM.zip › Figure4/Fig4C/Image_data/+KifC1_8h.tif]

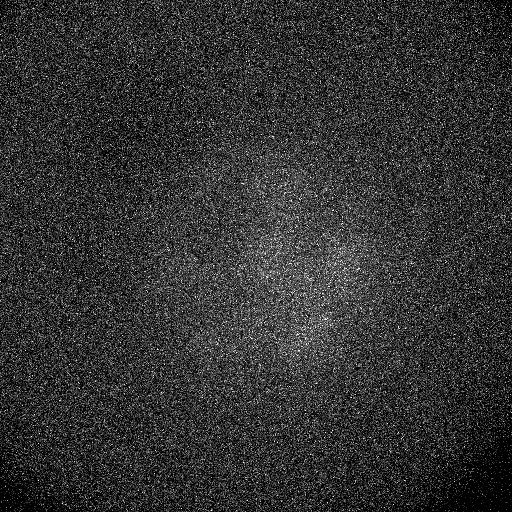

Supplement: Supplementary file 10 — Source data Fig. 4 [file 44318_2025_461_MOESM10_ESM.zip › Figure4/Fig4C/Image_data/Control_0h.tif]

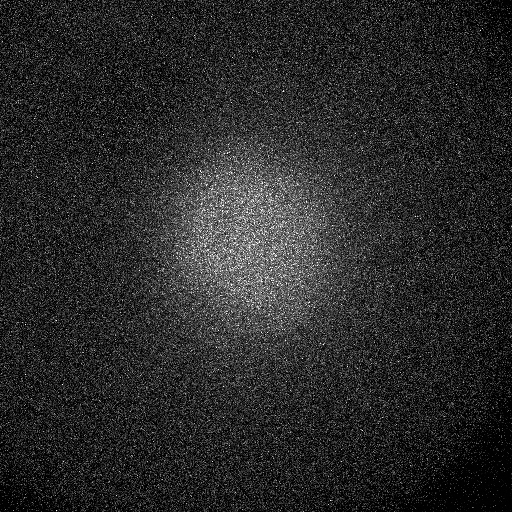

Supplement: Supplementary file 10 — Source data Fig. 4 [file 44318_2025_461_MOESM10_ESM.zip › Figure4/Fig4C/Image_data/Control_2h.tif]

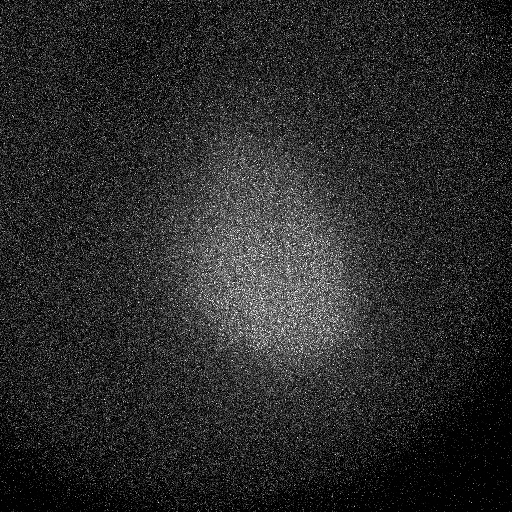

Supplement: Supplementary file 10 — Source data Fig. 4 [file 44318_2025_461_MOESM10_ESM.zip › Figure4/Fig4C/Image_data/Control_4h.tif]

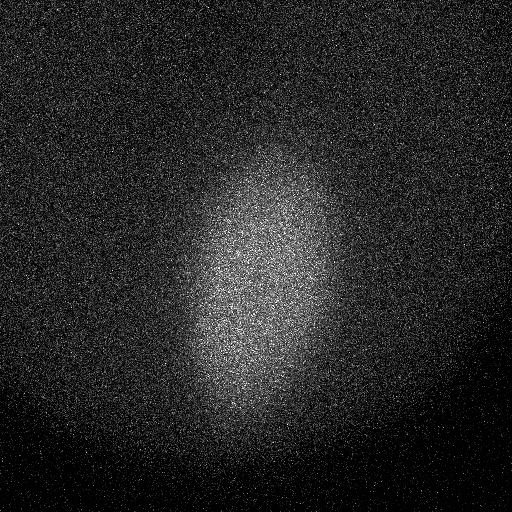

Supplement: Supplementary file 10 — Source data Fig. 4 [file 44318_2025_461_MOESM10_ESM.zip › Figure4/Fig4C/Image_data/Control_6h.tif]

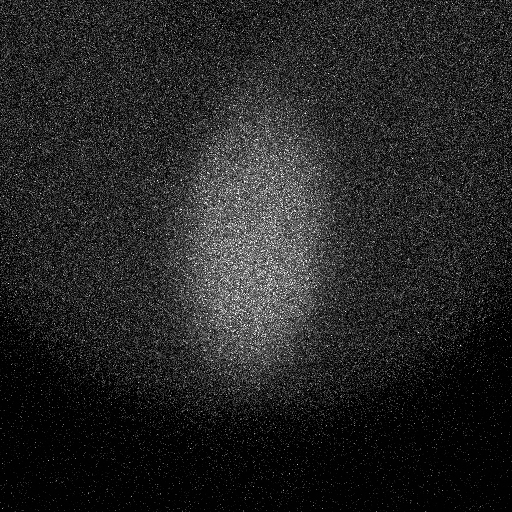

Supplement: Supplementary file 10 — Source data Fig. 4 [file 44318_2025_461_MOESM10_ESM.zip › Figure4/Fig4C/Image_data/Control_8h.tif]

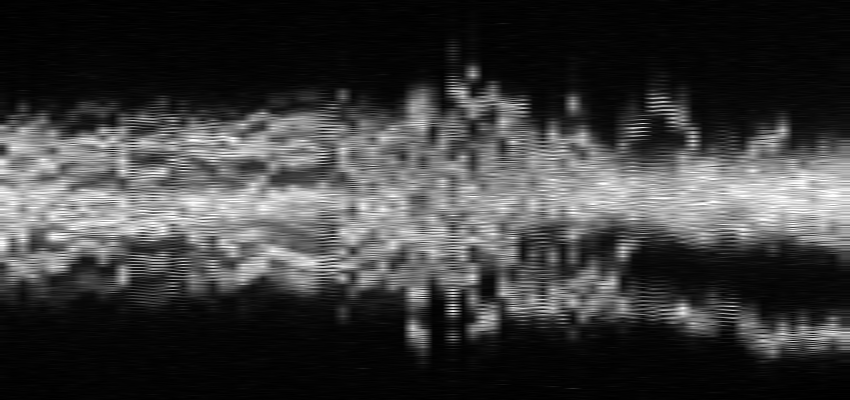

Supplement: Supplementary file 10 — Source data Fig. 4 [file 44318_2025_461_MOESM10_ESM.zip › Figure4/Fig4D/Image_data/Control.tif]

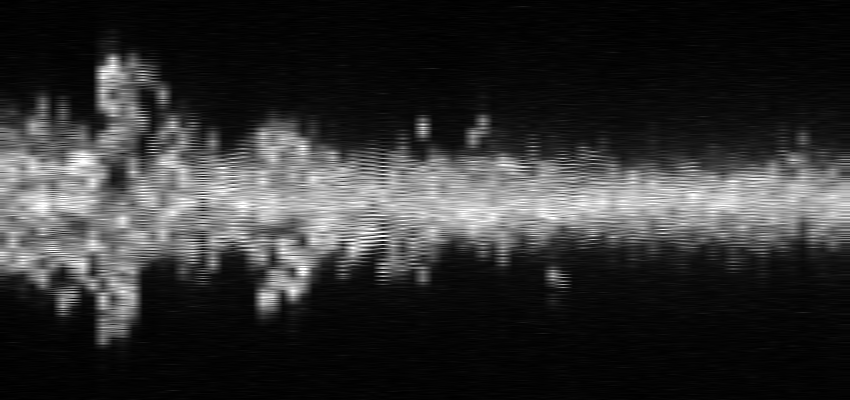

Supplement: Supplementary file 10 — Source data Fig. 4 [file 44318_2025_461_MOESM10_ESM.zip › Figure4/Fig4D/Image_data/KifC1.tif]

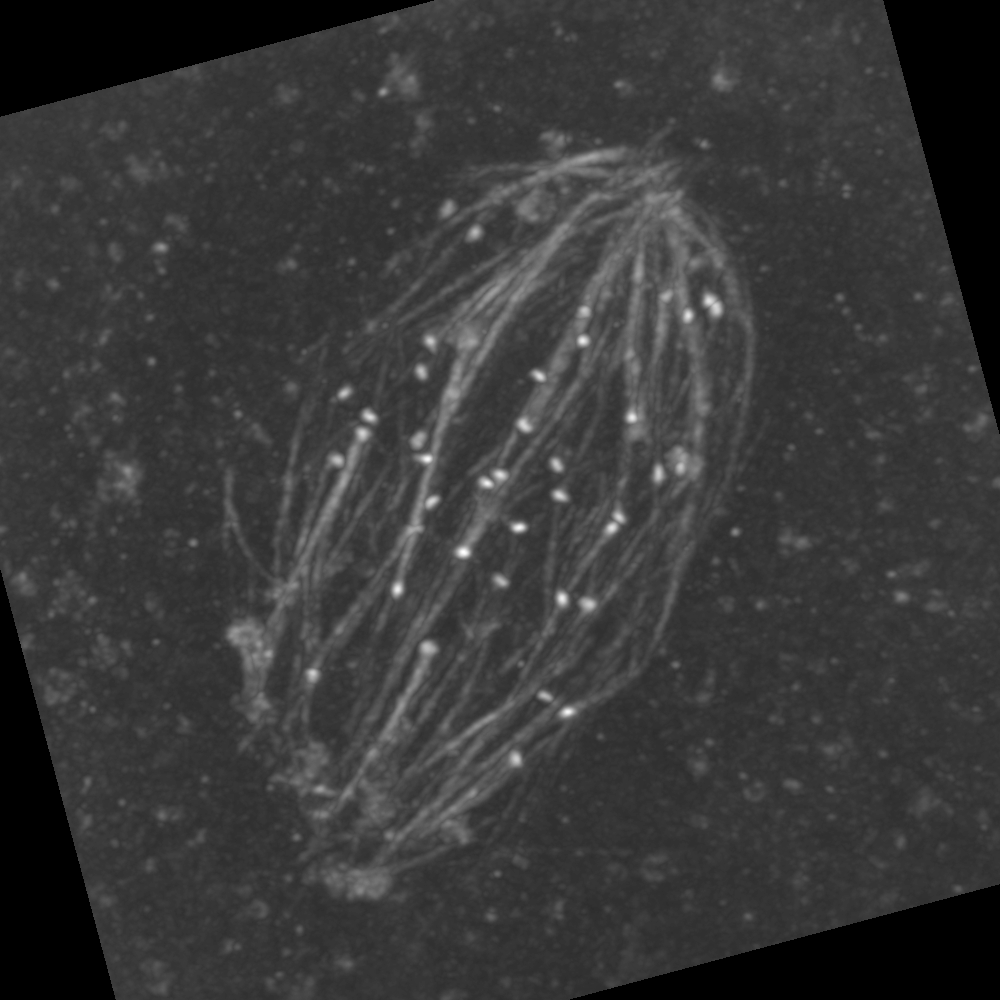

Supplement: Supplementary file 10 — Source data Fig. 4 [file 44318_2025_461_MOESM10_ESM.zip › Figure4/FIg4E/Image_data/Control.tif]

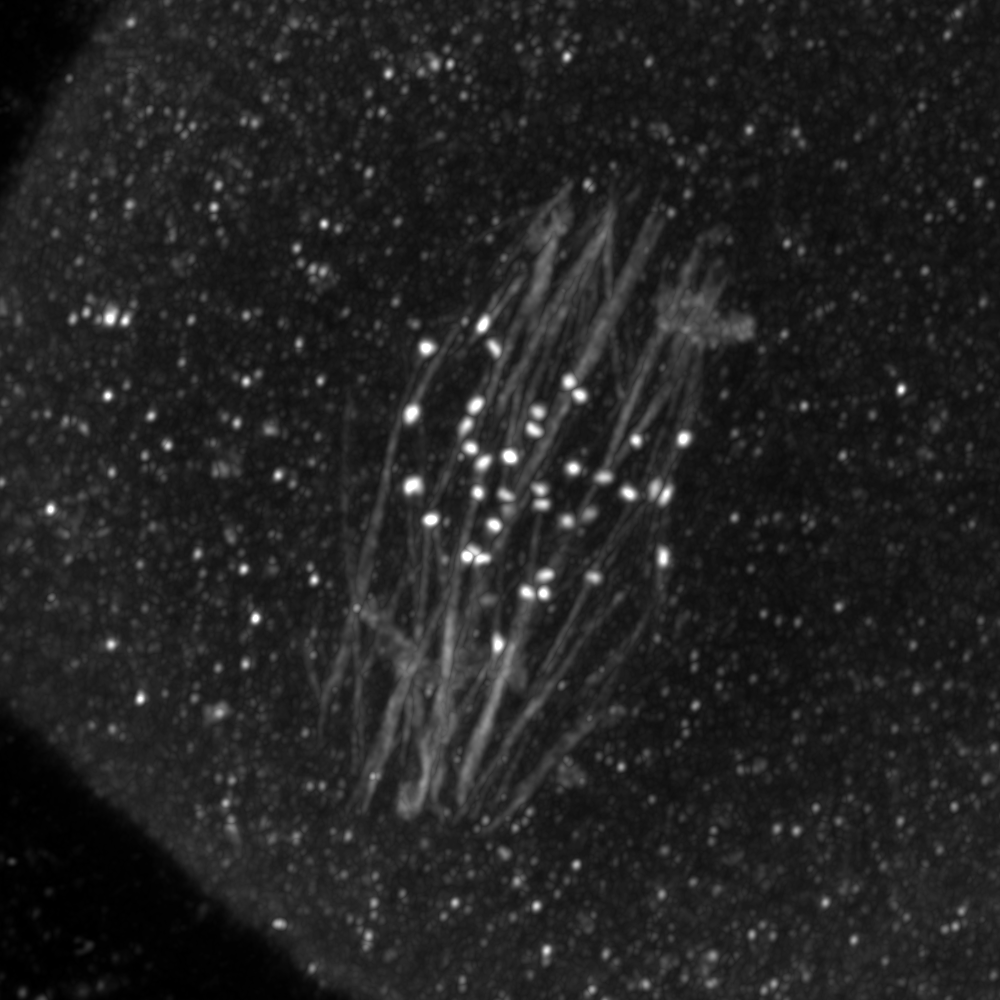

Supplement: Supplementary file 10 — Source data Fig. 4 [file 44318_2025_461_MOESM10_ESM.zip › Figure4/FIg4E/Image_data/KifC1.tif]
