# Supplementary material for: MPS1 promotes timely spindle bipolarization to prevent kinetochore-microtubule attachment errors in oocytes
Source: EMBO J. 2025 Jun 4;44(13):3794–823. doi: 10.1038/s44318-025-00461-w (PMC12214816; doi:10.1038/s44318-025-00461-w)
Supplement: Supplementary file 12 — Expanded View Figures [file 44318_2025_461_MOESM12_ESM.pdf]

Expanded View Figures

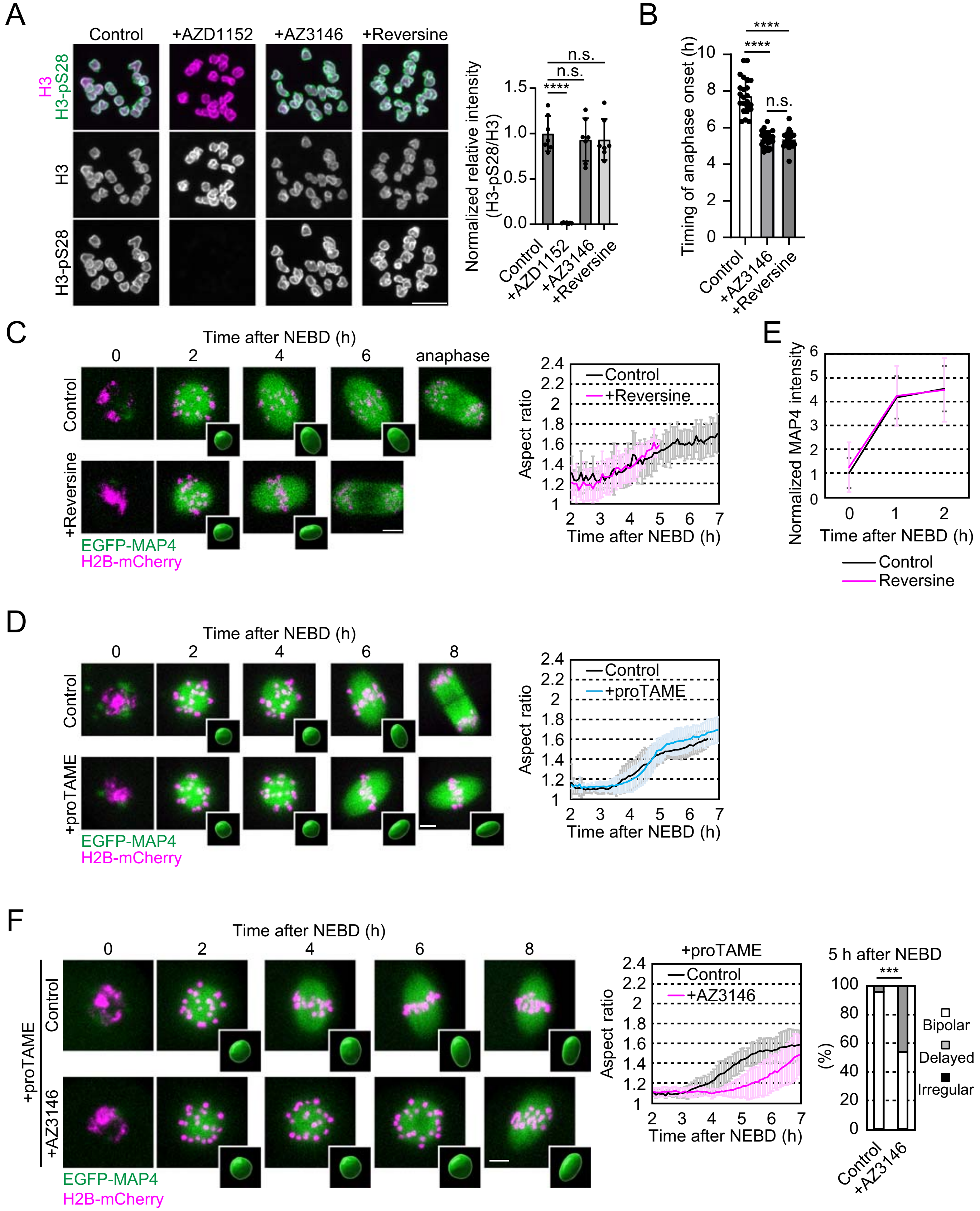

Figure EV1. MPS1 activity is required for efficient spindle bipolarization.

(**A**) Reversine or AZ3146 does not significantly inhibit Aurora B/C. Immunostaining of histone H3, H3-pS28 and Hoechst33342 (DNA) in oocytes treated with AZD1152 (Aurora B/C inhibitor), AZ3146, or reversine. Drugs were added to oocyte culture immediately after induction of meiotic resumption. Oocytes were fixed at 2 h after NEBD. Normalized relative intensities of H3-pS28 are shown (mean ± SD, $n = 7, 7, 7, 7$ oocytes. Three independent experiments were performed). n.s. not significant, ****$P = 0.0000000005$ by Tukey's multiple comparison test. Note that H3-pS28, an Aurora B/C target, was significantly reduced by AZD1142, but not by AZ3146 or reversine. (**B**) Reversine and AZ3146 accelerate anaphase onset. Timing of anaphase onset in oocytes treated with AZ3146 or reversine are shown (mean ± SD, $n = 23, 23, 23$ oocytes from three independent experiments). n.s. not significant, ****$P < 0.0000000001$ by Tukey's multiple comparison test. Oocytes treated with reversine or AZ3146 exhibited accelerated anaphase onset, consistent with spindle checkpoint defects caused by MPS1 inhibition (Hached et al, 2011; Yakoubi et al, 2017). (**C**) MPS1 inhibition does not delay spindle bipolarization but prematurely show anaphase spindle elongation. Live imaging of oocytes treated with reversine. Temporal changes in the aspect ratio of the spindle are shown (mean ± SD, $n = 8, 8$ oocytes). (**D**) ProTAME does not impair spindle bipolarization. Live imaging of oocytes treated with proTAME. Temporal changes in the aspect ratio of the spindle are shown (mean ± SD, $n = 9, 10$ oocytes. Three independent experiments were performed). (**E**) Initial microtubule nucleation is not significantly affected by MPS1 inhibition. Live imaging was performed on oocytes treated with proTAME and reversine (images are shown in Fig. 1B). Normalized EGFP-MAP4 intensities are shown (mean ± SD, $n = 26, 26$ oocytes from 4 independent experiments). (**F**) MPS1 inhibition delays spindle bipolarization in proTAME-treated oocytes. Live imaging of oocytes with proTAME and the MPS1 inhibitor AZ3146. Temporal changes in the aspect ratio of the spindle (mean ± SD, $n = 8, 8$ oocytes) and morphology classification at 5 h after NEBD ($n = 25, 26$ from 4 independent experiments) are shown. ***$P = 0.0008$ by Fisher's exact test for "bipolar" groups. Scale bars, 10 µm. Source data are available online for this figure.

 © The Author(s)

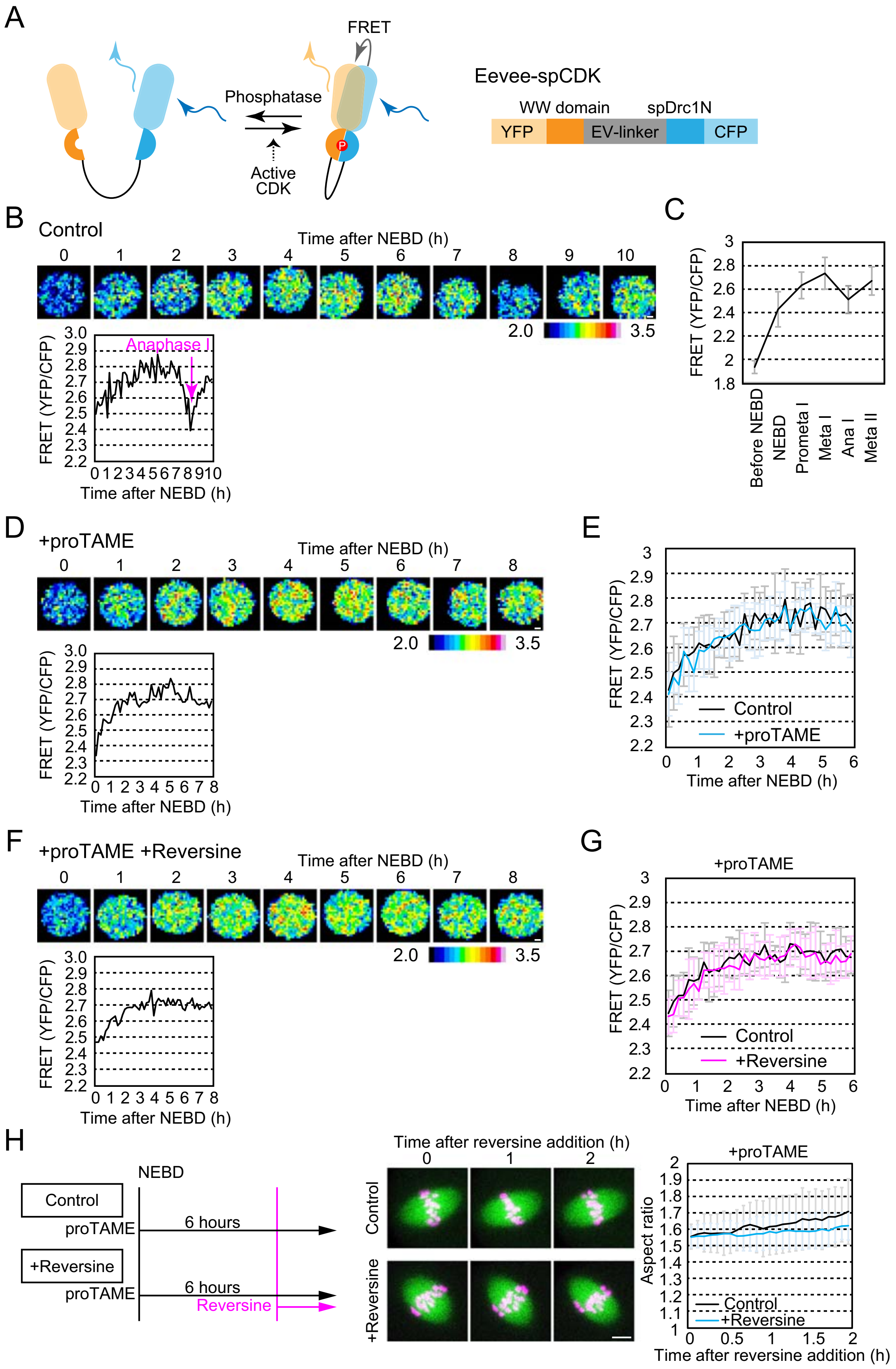
A
FRET
Phosphatase
Active
CDK
P
Eevee-spCDK
WW domain
spDrc1N
YFP
EV-linker
CFP
B
Control
Time after NEBD (h)
0 1 2 3 4 5 6 7 8 9 10
2.0
3.5
FRET (YFP/CFP)
Anaphase I
Time after NEBD (h)
C
FRET (YFP/CFP)
Before NEBD
NEBD
Prometa I
Meta I
Ana I
Meta II
D
+proTAME
Time after NEBD (h)
E
Control
+proTAME
F
+proTAME +Reversine
G
+proTAME
Control
+Reversine
H
NEBD
Control
proTAME
6 hours
+Reversine
Reversine
Time after reversine addition (h)
+Reversine
Aspect ratio
Time after reversine addition (h)

Figure EV2. ProTAME or additional reversine treatment does not affect CDK1 activity dynamics during prometaphase and metaphase.

(**A**) Schematic illustration of the CDK biosensor Eevee-SpCDK. (**B**, **C**) Temporal dynamics of the CDK1 activity. The CDK1 activity sensor Eevee-spCDK was monitored with live imaging. FRET (YFP/CFP) ratio images are shown. The plot in (**B**) shows the cytoplasmic FRET ratio in an oocyte. The plot in C shows the cytoplasmic FRET ratio at "Before NEBD" (0.5 h before NEBD), "NEBD", "Prometa I" (2 h after NEBD), "Meta I" (1 h before anaphase onset), "Ana I" (1 h after anaphase onset), and "Meta II" (3 h after anaphase onset) (mean ± SD, $n = 9$ oocytes from three independent experiments). Note that the Eevee-spCDK FRET dynamics is consistent with the CDK1 activity dynamics that increases upon M-phase entry (NEBD), gradually increases through prometaphase I and metaphase I, decreases at anaphase I, and then increases again at metaphase II (Choi et al, 1991; Davydenko et al, 2013). (**D**, **E**) CDK1 activity dynamics in proTAME-treated oocytes. FRET (YFP/CFP) ratio images are shown. The plots show the data from an oocyte (in **D**) and multiple oocytes (in **E**, mean ± SD, $n = 9$, 9 oocytes). Three independent experiments were performed. (**F**, **G**) CDK1 activity dynamics in oocytes treated with proTAME and reversine. Images and plots are shown as in (**D**, **E**) (mean ± SD, $n = 12$, 12 oocytes). Three independent experiments were performed. (**H**) MPS1 is not required for bipolar spindle maintenance. Live imaging of oocytes expressing EGFP-MAP4 (microtubules, green) and H2B-mCherry (chromosomes, magenta). Oocytes were cultured in the presence of proTAME. Reversine was added at 6 h after NEBD (metaphase I). Temporal changes in the aspect ratio of the spindle after reversine addition (mean ± SD, $n = 15$, 13 oocytes from 3 independent experiments) are shown. Scale bars, 10 µm.

 © The Author(s)

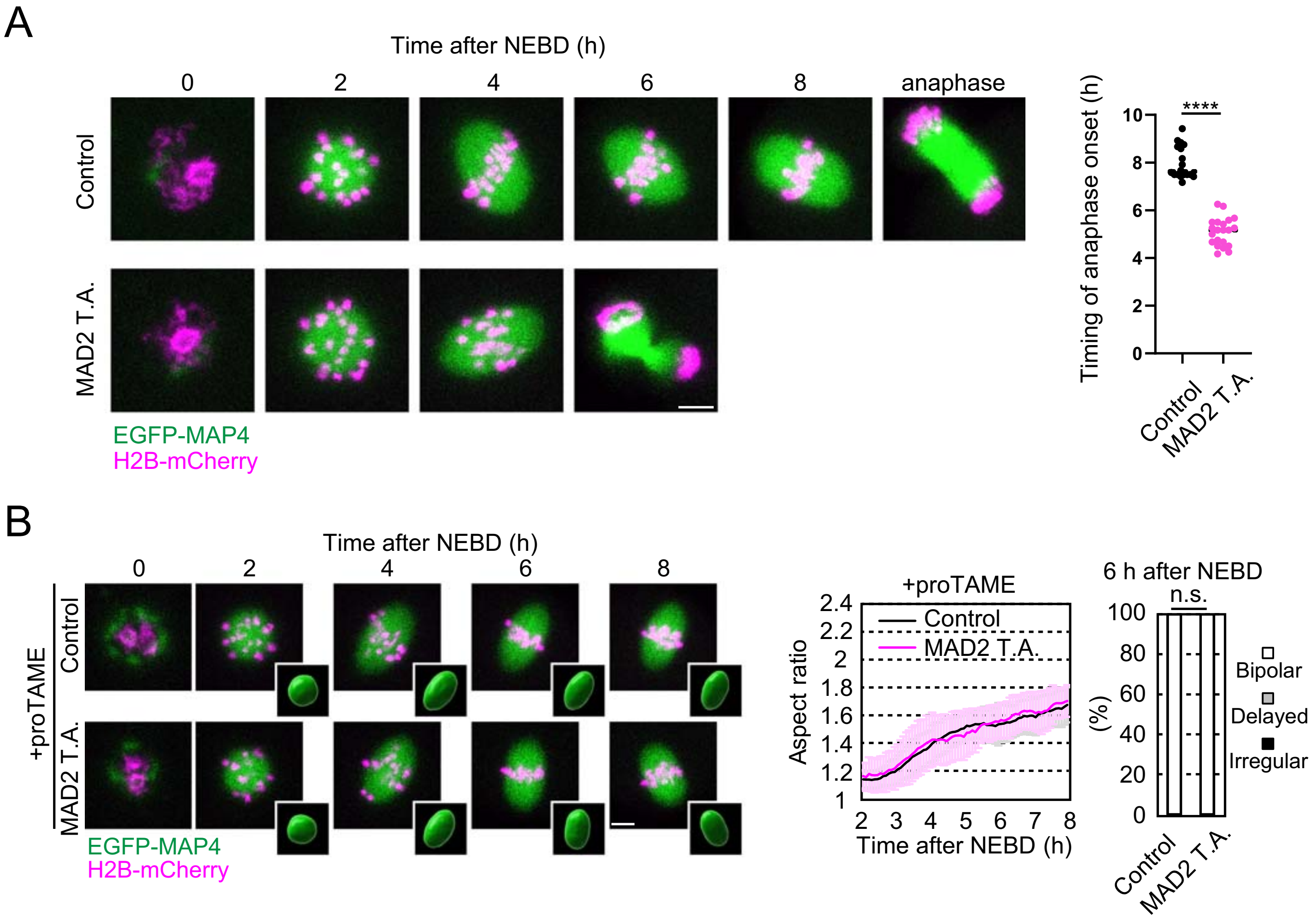

Figure EV3. Defective spindle checkpoint does not delay spindle bipolarization.

(**A**) MAD2 TRIM-Away accelerates anaphase onset in mouse oocytes. Representative z-projection images of EGFP-MAP4 (spindle, green) and H2B-mCherry (chromosome, magenta) are shown. MAD2 antibody was co-injected with TRIM21 mRNA for MAD2 TRIM-Away (T.A.). Timing of anaphase onset after NEBD are shown ($n = 18$, 21 oocytes from 3 independent experiments). ****$P = 0.00000000003$ by two-tailed unpaired Mann–Whitney test. (**B**) MAD2 is not required for spindle bipolarization in mouse oocytes. Live imaging of oocytes with proTAME. Temporal changes in the aspect ratio of the spindle (mean ± SD, $n = 14$, 14 oocytes) and morphology classification at 6 h after NEBD ($n = 27$, 28 oocytes) are shown. Four independent experiments were performed. n.s., not significant by Fisher's exact test for "bipolar" groups. Scale bars, 10 µm.

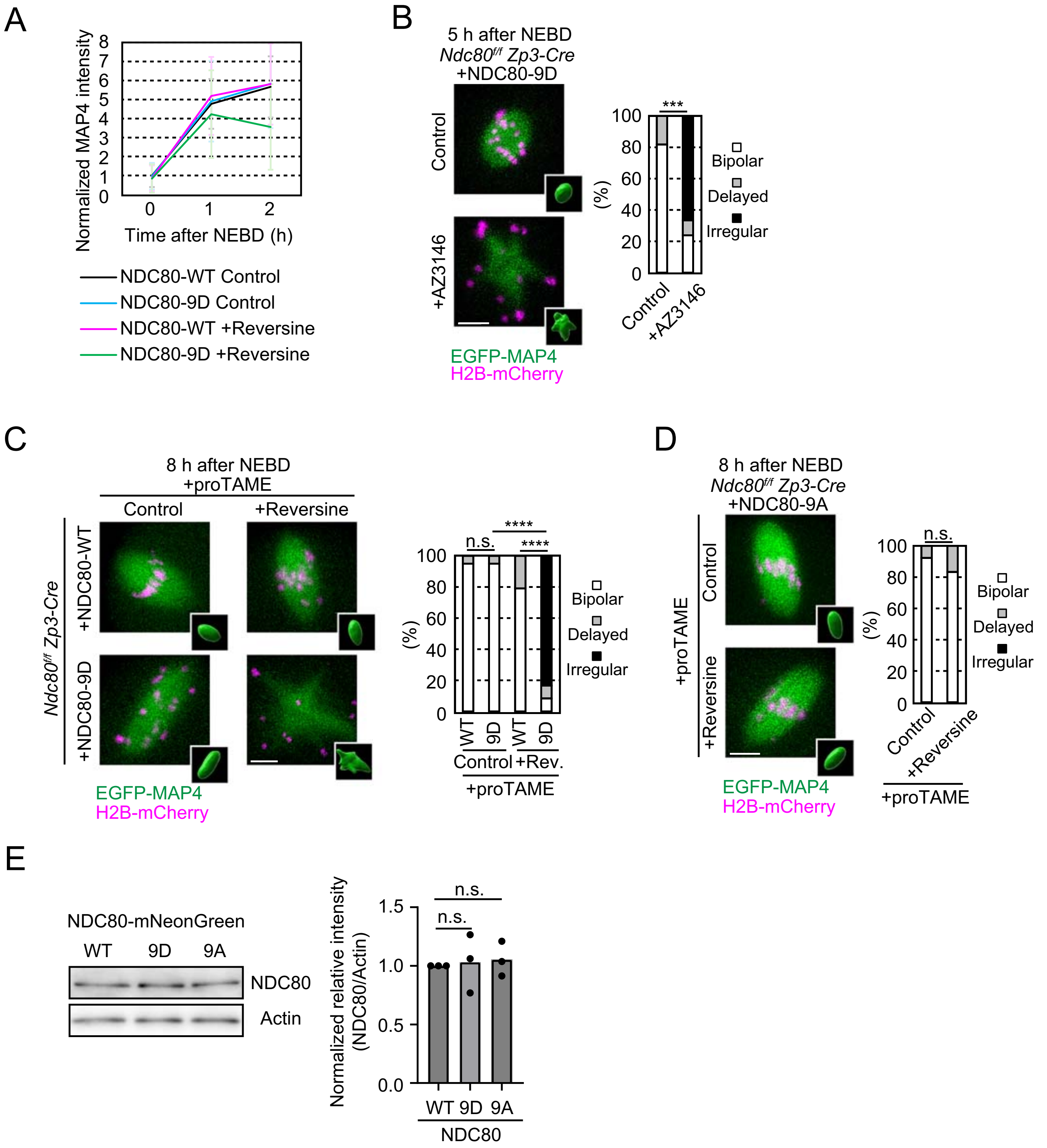
A
Normalized MAP4 intensity
Time after NEBD (h)
NDC80-WT Control
NDC80-9D Control
NDC80-WT +Reversine
NDC80-9D +Reversine
B
5 h after NEBD
Ndc80f/f Zp3-Cre
+NDC80-9D
Control
+AZ3146
EGFP-MAP4
H2B-mCherry
***
(%)
Bipolar
Delayed
Irregular
C
8 h after NEBD
+proTAME
Control
+Reversine
Ndc80f/f Zp3-Cre
+NDC80-WT
+NDC80-9D
n.s.
****
Control
+Rev.
D
8 h after NEBD
Ndc80f/f Zp3-Cre
+NDC80-9A
E
NDC80-mNeonGreen
WT 9D 9A
NDC80
Actin
Normalized relative intensity (NDC80/Actin)
NDC80

 © The Author(s)

Figure EV4. MPS1 activity is required for spindle bipolarization in the absence of stable kinetochore–microtubule attachment.

(**A**) MPS1 is required for microtubule maintenance in the absence of stable kinetochore–microtubule attachments. Live imaging was performed on *Ndc80*$^{f/f}$ *Zp3-Cre* oocytes expressing NDC80-WT/-9D and treated with reversine, using the microtubule marker EGFP-MAP4 (images are shown in Fig. 1C, D). Normalized EGFP-MAP4 intensities are shown (mean ± SD, $n = 21$, 25, 25, 23 from 3 independent experiments) are shown. (**B**) MPS1 inhibition impairs spindle bipolarization in NDC80-9D oocytes. Live imaging of *Ndc80*$^{f/f}$ *Zp3-Cre* oocytes expressing EGFP-MAP4 (spindle, green), H2B-mCherry (chromosome, magenta) and NDC80-9D with the MPS1 inhibitor AZ3146. Z-projection and 3D-reconstruction images at 5 h after NEBD are shown. Spindle morphology classification is shown ($n = 22$, 21 oocytes from three independent experiments). ***$P = 0.0002$. by Fisher's exact test for "bipolar" groups. (**C**) MPS1 inhibition impairs spindle bipolarization in NDC80-9D oocytes even after metaphase arrest. Live imaging of *Ndc80*$^{f/f}$ *Zp3-Cre* oocytes expressing NDC80-WT/-9D in the presence of proTAME. Z-projection and 3D-reconstruction images at 8 h after NEBD are shown. *Ndc80*$^{f/f}$ *Zp3-Cre* oocytes expressing NDC80-WT/-9D were analyzed as in (**B**) ($n = 20$, 20, 24, 24 oocytes from three independent experiments). n.s. not significant, ****$P = 0.000000010$ (9D control vs 9D revesine), 0.000001 (WT reversine vs 9D reversine) by Fisher's exact test for "bipolar" groups. (**D**) MPS1 inhibition does not impair spindle bipolarization in NDC80-9A oocytes. Z-projection and 3D-reconstruction images at 8 h after NEBD are shown. *Ndc80*$^{f/f}$ *Zp3-Cre* oocytes expressing NDC80-9A were analyzed as in (**B**) ($n = 26$, 24 oocytes from 3 independent experiments). n.s., not significant by Fisher's exact test for "bipolar" groups. (**E**) Protein levels of the mutant forms of NDC80. Oocytes at the GV stage were used for the Western blotting of NDC80-WT/9D/9A-mNeonGreen and actin. Normalized relative intensities from three independent experiments are shown. n.s., not significant by Tukey's multiple comparison test.

© The Author(s)

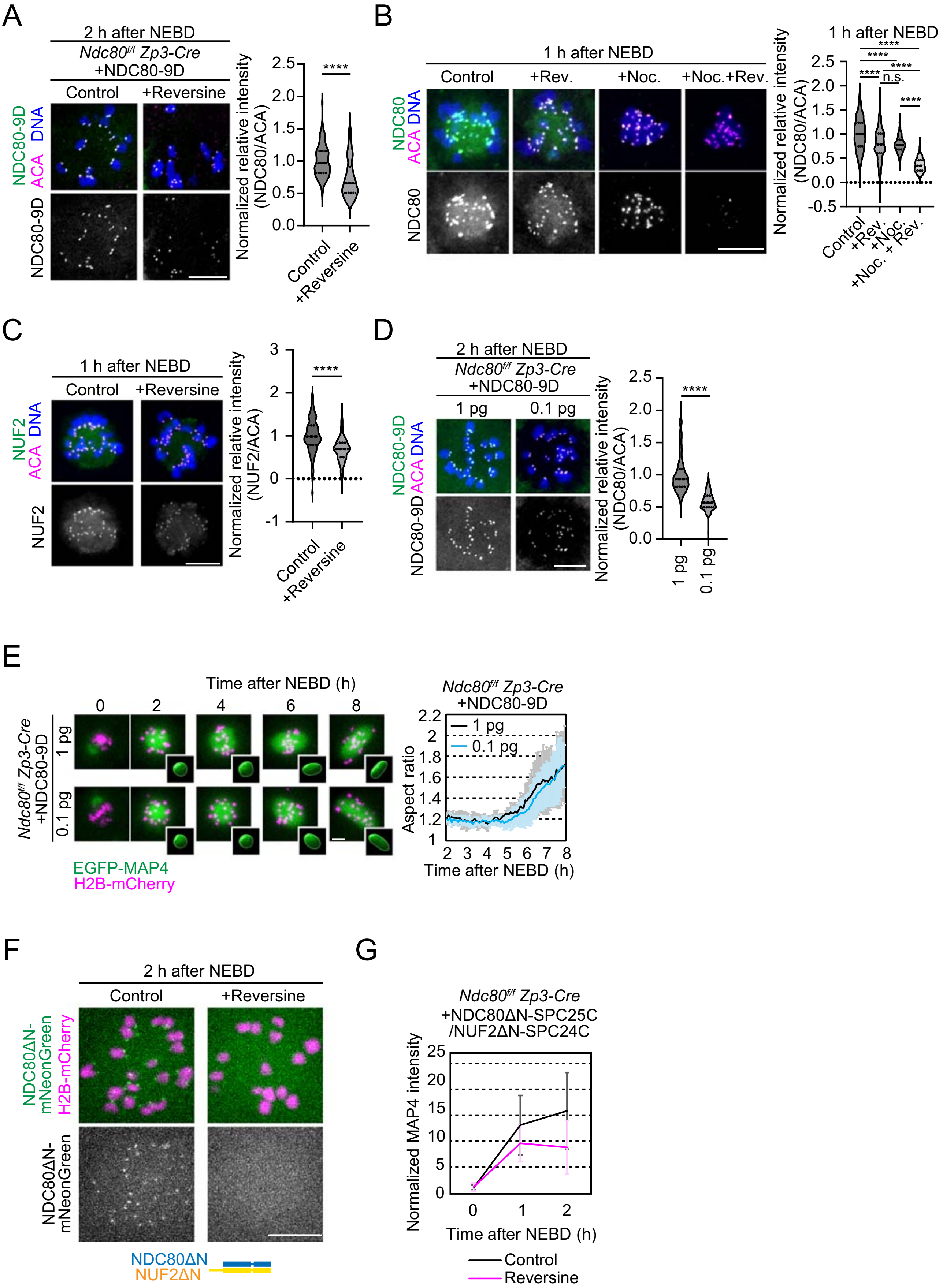
A
2 h after NEBD
Ndc80f/f Zp3-Cre
+NDC80-9D
Control
+Reversine
NDC80-9D ACA DNA
NDC80-9D
Normalized relative intensity (NDC80/ACA)
****
B
1 h after NEBD
Control
+Rev.
+Noc.
+Noc.+Rev.
NDC80 ACA DNA
NDC80
Normalized relative intensity (NDC80/ACA)
n.s.
C
1 h after NEBD
Control
+Reversine
NUF2 ACA DNA
NUF2
Normalized relative intensity (NUF2/ACA)
D
2 h after NEBD
Ndc80f/f Zp3-Cre
+NDC80-9D
1 pg
0.1 pg
NDC80-9D ACA DNA
NDC80-9D
Normalized relative intensity (NDC80/ACA)
E
Time after NEBD (h)
Ndc80f/f Zp3-Cre
+NDC80-9D
1 pg
0.1 pg
EGFP-MAP4
H2B-mCherry
Aspect ratio
Time after NEBD (h)
F
2 h after NEBD
Control
+Reversine
NDC80ΔN-mNeonGreen H2B-mCherry
NDC80ΔN-mNeonGreen
NDC80ΔN
NUF2ΔN
G
Ndc80f/f Zp3-Cre
+NDC80ΔN-SPC25C
/NUF2ΔN-SPC24C
Normalized MAP4 intensity
Time after NEBD (h)
Control
Reversine

 © The Author(s)

Figure EV5. MPS1 promotes kinetochore localization of NDC80 and NUF2.

(**A**) MPS1 inhibition decreases NDC80-9D localization at kinetochores. Immunostaining of NDC80-9D-GFP, ACA (kinetochores), and Hoechst33342 (DNA) in oocytes treated with reversine. Normalized relative intensities of NDC80-9D-GFP are shown (median and quartiles, $n = 280$, 174 kinetochores from 7, 7 oocytes). Three independent experiments were performed. ****$P < 0.0000000001$ by two-tailed unpaired Mann–Whitney test. (**B**) MPS1 inhibition decreases NDC80 localization at kinetochores. Immunostaining of anti-NDC80, ACA (kinetochores), and Hoechst33342 (DNA) in oocytes treated with reversine and/or nocodazole at 1 h after NEBD. Normalized relative intensities of NDC80 are shown (median and quartiles, $n = 240$, 240, 240, 232 kinetochores from 6, 6, 6, 6 oocytes). Three independent experiments were performed. n.s. not significant, ****$P < 0.0000000001$ by Tukey's multiple comparison test. (**C**) MPS1 inhibition decreases NUF2 localization at kinetochores. Immunostaining of NUF2-HA, ACA (kinetochores), and Hoechst33342 (DNA) in oocytes treated with reversine. Normalized relative intensities of NUF2-HA are shown (median and quartiles, $n = 280$, 280 kinetochores from 7, 7 oocytes). Three independent experiments were performed. ****$P < 0.0000000001$ by two-tailed unpaired Student's *t* test. (**D**) Titration of NDC80-9D. Immunostaining of NDC80-9D-GFP, ACA (kinetochores) and Hoechst33342 (DNA) in oocytes expressing 1 pg or 0.1 pg mRNA of NDC80-9D-GFP. Normalized relative intensities of NDC80-9D-GFP are shown (median and quartiles, $n = 280$, 200 kinetochores of 7, 5 oocytes). Two independent experiments were performed. ****$P < 0.0000000001$ by two-tailed unpaired Mann–Whitney test. (**E**) Reduced NDC80-9D can support spindle bipolarization. Live imaging of *Ndc80$^{f/f}$ Zp3-Cre* oocytes expressing EGFP-MAP4 (spindle, green), H2B-mCherry (chromosome, magenta), and NDC80-9D. Temporal changes in the aspect ratio of the spindle are shown (mean ± SD, $n = 6$, 7 oocytes). Three independent experiments were performed. (**F**) MPS1 inhibition delocalizes NDC80ΔN from kinetochores. *Ndc80$^{f/f}$ Zp3-Cre* oocytes expressing NDC80ΔN-mNeonGreen, NUF2ΔN, and H2B-mCherry treated with reversine were imaged. Three independent experiments were performed. (**G**) MPS1 is required for microtubule maintenance in the absence of stable kinetochore–microtubule attachments. Live imaging was performed on *Ndc80$^{f/f}$ Zp3-Cre* oocytes expressing NDC80ΔN-SPC25C and NUF2ΔN-SPC24C and treated with reversine, using the microtubule marker EGFP-MAP4 (images are shown in Fig. 2D). Normalized EGFP-MAP4 intensities are shown (mean ± SD, $n = 26$, 26 from three independent experiments). The experiment was performed in the presence of proTAME. Scale bars, 10 μm.

© The Author(s)

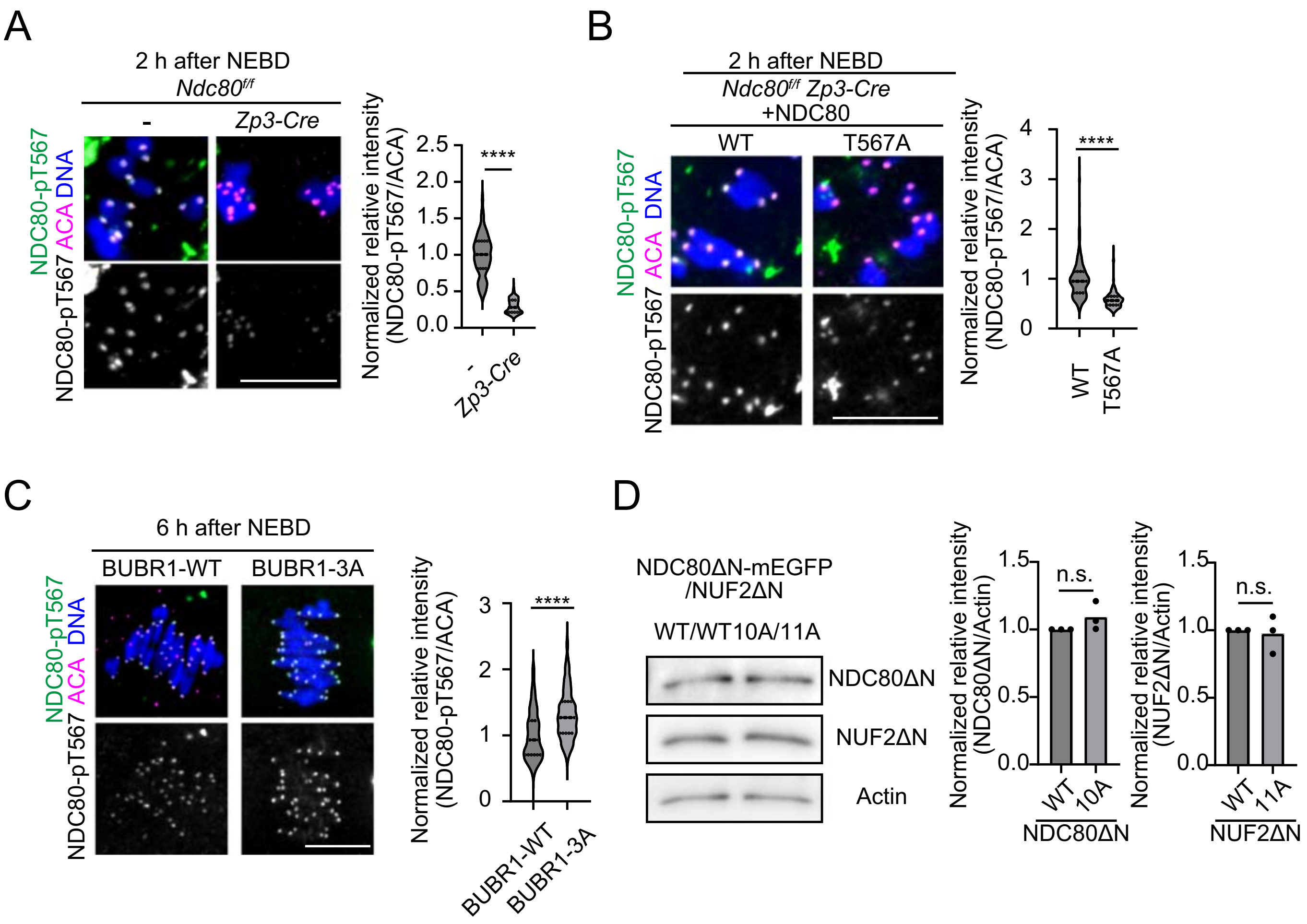

Figure EV6. MPS1 promotes spindle bipolarization via the C-terminal domains of NDC80-NUF2 during prometaphase.

(**A**) Phospho-antibody specificity. *Ndc80*$^{f/f}$ *Zp3-Cre* oocytes were immunostained with anti-phospho-NDC80-T567 antibody, ACA (kinetochores), and Hoechst33342 (DNA). Control oocytes were without *Zp3-Cre*. Normalized relative intensities of phospho-NDC80-T567 are shown (median and quartiles, $n = 199$, 168 kinetochores from 5, 5 oocytes). Three independent experiments were performed. ****$P < 0.0000000001$ by two-tailed unpaired Mann–Whitney test. (**B**) Phospho-antibody specificity. *Ndc80*$^{f/f}$ *Zp3-Cre* oocytes expressing NDC80-WT/-T567A were immunostained with anti-phospho-NDC80-T567, ACA (kinetochores), and Hoechst33342 (DNA). Normalized relative intensities of NDC80-pT567 are shown (median and quartiles, $n = 240$, 240 kinetochores from 6, 6 oocytes). Three independent experiments were performed. ****$P < 0.0000000001$ by two-tailed unpaired Mann–Whitney test. (**C**) NDC80-T567 phosphorylation increases in BUBR1-3A-overexpressing oocytes. Oocytes expressing mEGFP-BUBR1-WT/3 A were immunostained with anti-phosphorylated NDC80 at T567 (NDC80-pT567), ACA (kinetochores), and Hoechst33342 (DNA). Normalized relative intensities of NDC80-pT567 are shown (median and quartiles, $n = 240$, 240 kinetochores from 6, 6 oocytes). Three independent experiments were performed. ****<0.0000000001 by two-tailed unpaired Mann–Whitney test. (**D**) Protein levels of the mutant forms of NDC80ΔN and NUF2ΔN. Oocytes at the GV stage were used for the Western blotting of NDC80ΔN-WT/10A-mEGFP, NUF2ΔN-WT/11 A and actin. Normalized relative intensities from three independent experiments are shown. n.s., not significant by two-tailed unpaired Mann–Whitney test. Scale bars, 10 μm.

© The Author(s)

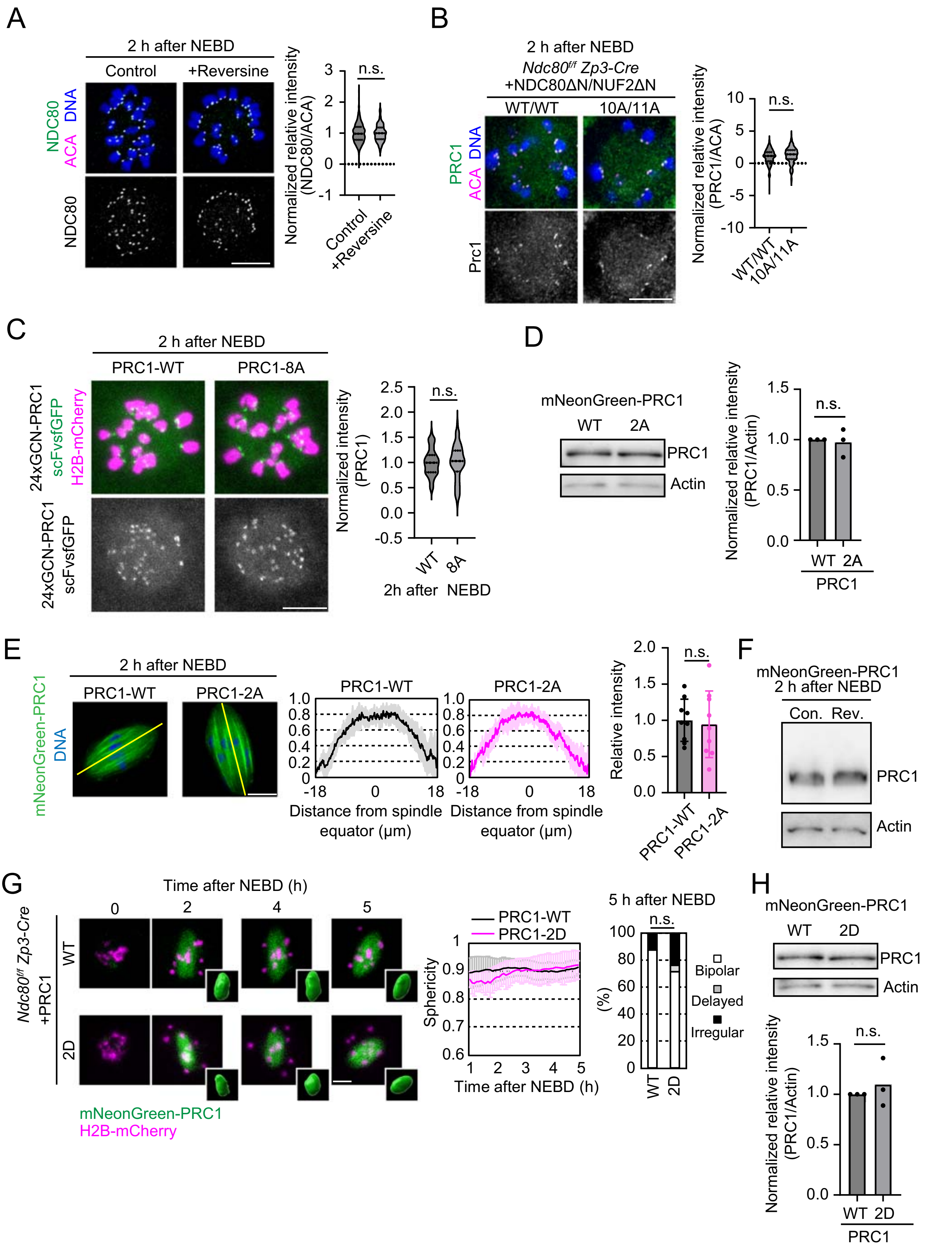
A
2 h after NEBD
Control
+Reversine
NDC80 ACA DNA
NDC80
Normalized relative intensity (NDC80/ACA)
n.s.
B
2 h after NEBD
Ndc80f/f Zp3-Cre
+NDC80ΔN/NUF2ΔN
WT/WT
10A/11A
PRC1 ACA DNA
Prc1
Normalized relative intensity (PRC1/ACA)
C
2 h after NEBD
PRC1-WT
PRC1-8A
24xGCN-PRC1 scFvsfGFP H2B-mCherry
24xGCN-PRC1 scFvsfGFP
Normalized intensity (PRC1)
2h after NEBD
D
mNeonGreen-PRC1
WT
2A
PRC1
Actin
Normalized relative intensity (PRC1/Actin)
E
2 h after NEBD
PRC1-WT
PRC1-2A
mNeonGreen-PRC1 DNA
Distance from spindle equator (μm)
Relative intensity
F
mNeonGreen-PRC1
2 h after NEBD
Con.
Rev.
G
Time after NEBD (h)
Ndc80f/f Zp3-Cre +PRC1
WT
2D
mNeonGreen-PRC1
H2B-mCherry
PRC1-WT
PRC1-2D
Sphericity
5 h after NEBD
Bipolar
Delayed
Irregular
(%)
H
mNeonGreen-PRC1
WT
2D
PRC1
Actin
Normalized relative intensity (PRC1/Actin)

Figure EV7. MPS1 activity promotes kinetochore localization and spindle bipolarization activity of PRC1.

(**A**) NDC80 is not significantly decreased in MPS1-inhibited oocytes at 2 h after NEBD. Immunostaining of reversine-treated oocytes with anti-NDC80, ACA (kinetochores), and Hoechst33342 (DNA). Normalized relative intensities of NDC80 are shown (median and quartiles, $n = 240$, 240 kinetochores from 6, 6 oocytes). Three independent experiments were performed. n.s., not significant by two-tailed unpaired Student's t test. (**B**) PRC1 can be recruited to kinetochores by phospho-mutant NDC80ΔN and NUF2ΔN. Immunostaining of *Ndc80*$^{f/f}$ *Zp3-Cre* oocytes expressing NDC80ΔN-WT/-10A and NUF2ΔN-WT/-11A with anti-PRC1, ACA (kinetochores), and Hoechst33342 (DNA). Normalized relative intensities of PRC1 are shown (median and quartiles, $n = 200$, 200 kinetochores from 5, 5 oocytes). Three independent experiments were performed. n.s., by two-tailed unpaired Student's *t* test. (**C**) Phospho-mutant PRC1 can localize to kinetochores. Oocytes expressing 24xGCN-PRC1-WT/-8A, scFv-sfGFP, and H2B-mCherry were imaged. Relative intensities of PRC1-WT or -8A at kinetochores are shown (median and quartiles, $n = 80$, 80 kinetochores from 4, 4 oocytes). Three independent experiments were performed. n.s., not significant by two-tailed unpaired Mann–Whitney test. (**D**) Protein levels of the mutant forms of PRC1. Oocytes at the GV stage were used for the Western blotting of mNeonGreen-PRC1-WT/2 A and actin. Normalized relative intensities from 3 independent experiments are shown. n.s., not significant by two-tailed unpaired Mann–Whitney test. (**E**) PRC1-2A preferentially localizes to the middle region of the spindle. Oocytes expressing mNeonGreen-PRC1-WT/2 A were fixed. Images of mNeonGreen-PRC1 and Hoechst33342 (DNA) are shown. In each oocyte, intensities of PRC1-WT or −2A along the spindle axis (yellow line) were normalized with their maximum intensity (mean ± SD, $n = 9$, 9 oocytes from three independent experiments). PRC1 intensities on the spindle were compared between PRC1-WT and −2A. n.s., not significant by two-tailed unpaired Mann–Whitney test. (**F**) No detectable MPS1-dependent band shifts of PRC1 in oocyte extracts. Oocytes expressing mNeonGreen-PRC1 were cultured in the presence of reversine. Oocytes at prometaphase (2 h after NEBD) were used for Phos-tag SDS-PAGE followed by Western blotting. (**G**) PRC1-2D can rescue spindle bipolarization in *Ndc80*-deleted oocytes. Live imaging of *Ndc80*$^{f/f}$ *Zp3-Cre* oocytes expressing mNeonGreen-PRC1-WT/2D (T578 and S583 substituted to aspartic acid, green) and H2B-mCherry (chromosome, magenta). Insets show 3D reconstructed images. Temporal changes in the sphericity of the spindle (mean ± SD, $n = 14$, 13 oocytes from two independent experiments) and morphology classification at 5 h after NEBD ($n = 24$, 21 oocytes from three independent experiments) are shown. n.s., not significant by Fisher's exact test for "bipolar" groups. (**H**) Protein levels of the mutant forms of PRC1. Oocytes at the GV stage were used for the Western blotting of mNeonGreen-PRC1-WT/2D and actin. Normalized relative intensities from three independent experiments are shown. n.s., not significant by two-tailed unpaired Mann–Whitney test. Scale bars, 10 µm.

 © The Author(s)

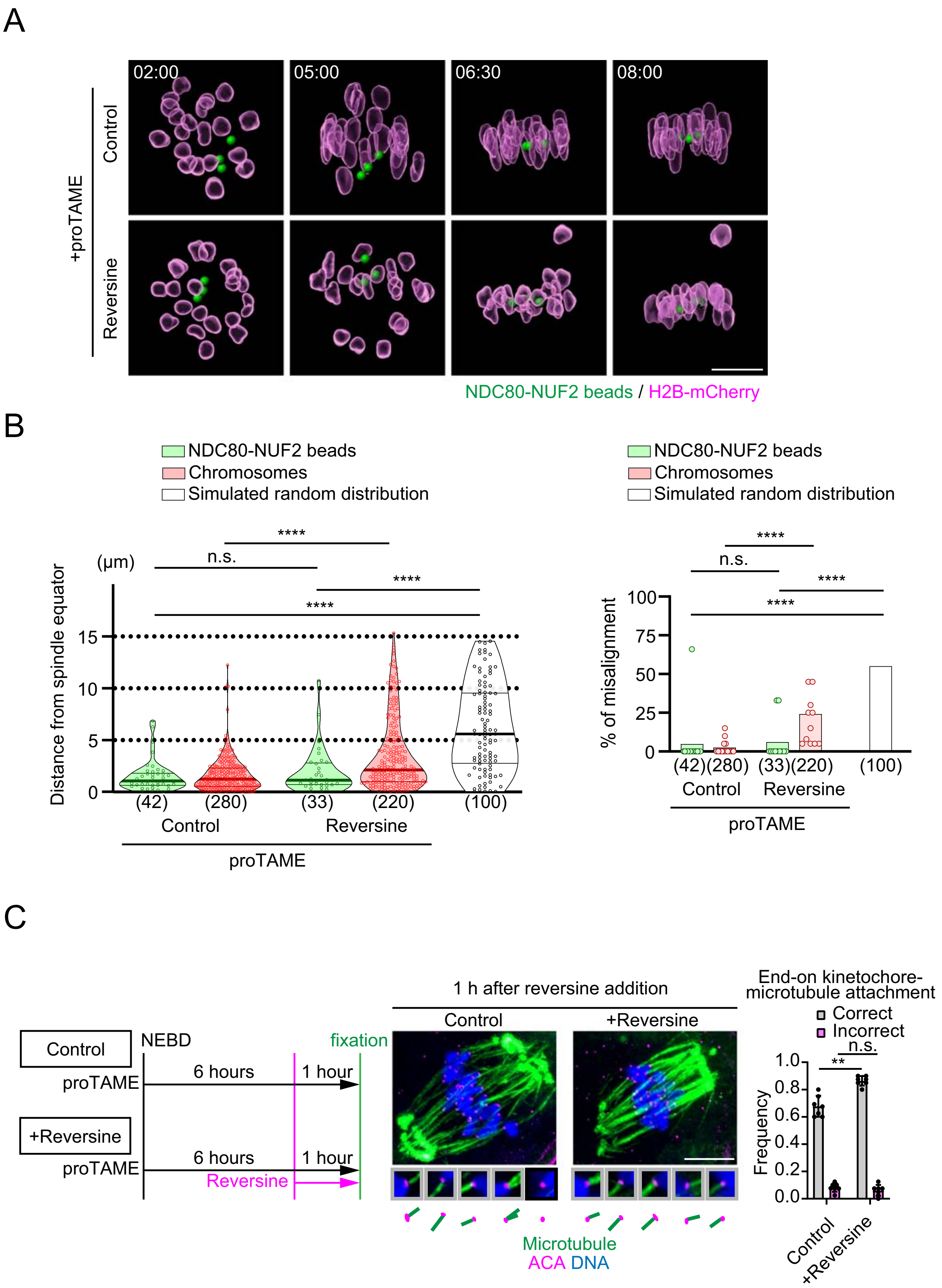
A
02:00
05:00
06:30
08:00
+proTAME
Control
Reversine
NDC80-NUF2 beads / H2B-mCherry
B
NDC80-NUF2 beads
Chromosomes
Simulated random distribution
(µm)
Distance from spindle equator
n.s.
****
15
10
5
0
(42)
(280)
(33)
(220)
(100)
Control
Reversine
proTAME
% of misalignment
100
75
50
25
C
Control
+Reversine
NEBD
proTAME
6 hours
1 hour
fixation
Reversine
1 h after reversine addition
Microtubule
ACA DNA
End-on kinetochore-microtubule attachment
Correct
Incorrect
Frequency
1.0
0.8
0.6
0.4
0.2
0.0
**

Figure EV8. MPS1 is not required for NDC80-NUF2 microbead alignment.

(**A**) Live imaging of NDC80-NUF2-tethered beads in oocytes. Oocytes expressing NDC80-GFP, NUF2 and H2B-mCherry and carrying anti-GFP beads were cultured for meiosis I in the presence of proTAME and reversine. 3D reconstructed images of chromosomes (H2B-mCherry, magenta) and NDC80-NUF2 beads (NDC80-GFP, green) are shown. Time after NEBD (hours:minutes). (**B**) NDC80-NUF2 beads can align in MPS1-inhibited oocytes. (Left) The distances of NDC80-NUF2 beads and chromosomes from the spindle equator are shown ($n = 42$, 33 beads, 280, 220 chromosomes from 14, 11 oocytes in one of three experiments). Simulated random distribution of particles within a spindle-like ellipsoid (30 µm in length and 20 µm in width) is used as a reference. n.s., not significant, ****$P = 0.00000000169$ (control chromosome vs reversine chromosome), 0.00000000002 (control NDC80-NUF2 beads vs simulated random distribution), 0.00000013011 (reversine NDC80-NUF2 beads vs simulated random distribution) by Kruskal-Wallis test with Dunn's correction. (Right) Fraction of misaligned NDC80-NUF2 beads and chromosomes are shown. Beads and chromosomes positions >5 µm from the spindle equator are defined as "misalignment". n.s., not significant. ****$P = 0.000000000000039$ (control chromosome vs reversine chromosome), 0.0000000040 (control NDC80-NUF2 beads vs simulated random distribution), 0.00000019 (reversine NDC80-NUF2 beads vs simulated random distribution) by Fisher's exact test. (**C**) MPS1 inhibition after metaphase spindle establishment does not increase kinetochore–microtubule attachment errors. Oocytes were cultured in the presence of proTAME. Reversine was added at 6 h after NEBD (metaphase I). Oocytes were collected 1 h after the reversine addition, treated briefly with a cold buffer, and then immunostained for stable microtubules (green), kinetochores (magenta), and DNA (blue). Magnified images of end-on monopolar attachments (correct, gray frame) are shown (mean ± SD, $n = 7$, 7 oocytes from three independent experiments). n.s., not significant, **$P = 0.001166$ by two-tailed unpaired Mann–Whitney test. Scale bars, 10 µm.

 © The Author(s)
